# Identification of New Non-BBB Permeable Tryptophan Hydroxylase Inhibitors for Treating Obesity and Fatty Liver Disease

**DOI:** 10.3390/molecules27113417

**Published:** 2022-05-25

**Authors:** Suvarna H. Pagire, Haushabhau S. Pagire, Kun-Young Park, Eun Jung Bae, Kwang-eun Kim, Minhee Kim, Jihyeon Yoon, Saravanan Parameswaran, Jun-Ho Choi, Sungmi Park, Jae-Han Jeon, Jin Sook Song, Myung Ae Bae, In-Kyu Lee, Hail Kim, Jae Myoung Suh, Jin Hee Ahn

**Affiliations:** 1 Department of Chemistry, Gwangju Institute of Science and Technology, Gwangju 61005, Korea; shpagire@gist.ac.kr (S.H.P.); hspagire@gist.ac.kr (H.S.P.); helloimej@gist.ac.kr (E.J.B.); kmh0724@gist.ac.kr (M.K.); yjh1@jdbiosci.com (J.Y.); dr.p.saravanan.bi@gmail.com (S.P.); junhochoi@gist.ac.kr (J.-H.C.); 2 JD Bioscience, 208 beon-gil, Cheomdangwagi-ro, Buk-gu, Gwangju 61005, Korea; 3 Graduate School of Medical Science and Engineering, Korea Advanced Institute of Science and Technology, Daejeon 34141, Korea; pky1171@kaist.ac.kr (K.-Y.P.); werd2000@snu.ac.kr (K.-e.K.); hailkim@kaist.edu (H.K.); 4 Biomedical Science and Engineering Interdisciplinary Program, Korea Advanced Institute of Science and Technology, Daejeon 34141, Korea; 5 Department of Biotechnology and Bioinformatics, School of Life Sciences, JSS Academy of Higher Education and Research (JSS AHER), Mysuru 570015, India; 6 Leading-Edge Research Center for Drug Discovery and Development for Diabetes and Metabolic Disease, Kyungpook National University Hospital, Daegu 41404, Korea; smpark93@gmail.com (S.P.); ggoloo@hanmail.net (J.-H.J.); leei@knu.ac.kr (I.-K.L.); 7 Department of Internal Medicine, School of Medicine, Kyungpook National University, Kyungpook National University Chilgok Hospital, Daegu 41404, Korea; 8 Bio and Drug Discovery Division, Korea Research Institute of Chemical Technology, Daejeon 34141, Korea; jssong@krict.re.kr (J.S.S.); mbae@krict.re.kr (M.A.B.); 9 Department of Internal Medicine, School of Medicine, Kyungpook National University, Kyungpook National University Hospital, Daegu 41944, Korea

**Keywords:** tryptophan hydroxylase inhibitor, obesity, fatty liver, treatment

## Abstract

Serotonin (5-hydroxytryptophan) is a hormone that regulates emotions in the central nervous system. However, serotonin in the peripheral system is associated with obesity and fatty liver disease. Because serotonin cannot cross the blood-brain barrier (BBB), we focused on identifying new tryptophan hydroxylase type I (TPH1) inhibitors that act only in peripheral tissues for treating obesity and fatty liver disease without affecting the central nervous system. Structural optimization inspired by *para*-chlorophenylalanine (pCPA) resulted in the identification of a series of oxyphenylalanine and heterocyclic phenylalanine derivatives as TPH1 inhibitors. Among these compounds, compound **18i** with an *IC*_50_ value of 37 nM was the most active in vitro. Additionally, compound **18i** showed good liver microsomal stability and did not significantly inhibit CYP and Herg. Furthermore, this TPH1 inhibitor was able to actively interact with the peripheral system without penetrating the BBB. Compound **18i** and its prodrug reduced body weight gain in mammals and decreased in vivo fat accumulation.

## 1. Introduction

**Figure 1:**
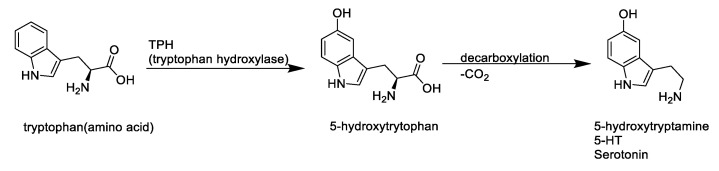
The synthesis of serotonin (5-hydroxytryptamine [5-HT]).

Serotonin (5-hydroxytryptamine [5-HT]) is an ancient biochemical that acts in the central nervous system and peripheral nervous system [1]. In the central nervous system, 5-HT is a neurotransmitter, which affects mood, appetite, sleep, and memory [2,3,4,5]. An imbalance in the serotonin system in the central nervous system has been implicated in a multitude of neuropsychiatric diseases. Recently, the new function regarding peripheral serotonin in energy homeostasis ranging from the endocrine regulation by gut-derived serotonin to the autocrine/paracrine regulation by adipocyte-derived serotonin was reported [6]. Serotonin plays an important role in metabolic regulation in peripheral tissues and is emerging as a possible target for anti-obesity and fatty liver disease treatment. Serotonin is synthesized from tryptophan by the sequential action of two enzymes, tryptophan hydroxylase (TPH) and aromatic amino acid decarboxylase with TPH catalyzing the rate-limiting step (Figure 1).

Oh et al. [7] reported that serotonin regulates white and brown adipose tissue function. Mice with inducible TPH1 KO in adipose tissues exhibit inhibition of lipogenesis in epididymal white adipose tissue (WAT), induction of browning in inguinal WAT, and activation of adaptive thermogenesis in brown adipose tissue (BAT).

The inability of serotonin to cross the Blood Brain Barriers (BBB) enforces the dualistic character of the serotonin system by creating two physiologically separated serotonin pools in the body. These characteristics of serotonin and KO study results prompted us to develop a TPH1 inhibitor that only acts in peripheral tissue for treating obesity and fatty liver disease. 

Several tryptophan hydroxylase type I (TPH1) inhibitors have been reported in the literature and patents. For example, *para*-chlorophenylalanine (pCPA), [8] also known as fenclonine, is a classic TPH1 inhibitor that has been developed to treat carcinoid syndrome. However, the in vitro binding activity of pCPA to TPH1 is very weak (*IC*_50_ > 50 µM). Additionally, pCPA causes side effects such as depression because it passes through the central nervous system. Recently, telotristat ethyl was marketed as a TPH1 inhibitor for carcinoid syndrome [9]. Scientists at Karos Pharmaceuticals have reported that spirocyclic TPH1 inhibitors such as telotristat ethyl can be used to treat Pulmonary Arterial Hypertension (PAH) and Inflammatory Bowel Disease (IBD) [10].

Herein, we attempt to identify new TPH1 inhibitors starting from pCPA by introducing a non-BBB permeable moiety. The design, synthesis, and biological evaluation of a diverse suite of phenylalanine derivatives for preventing obesity and fatty liver disease are presented.

## 2. Results and Discussion

### 2.1. Chemistry

**Scheme 1:**
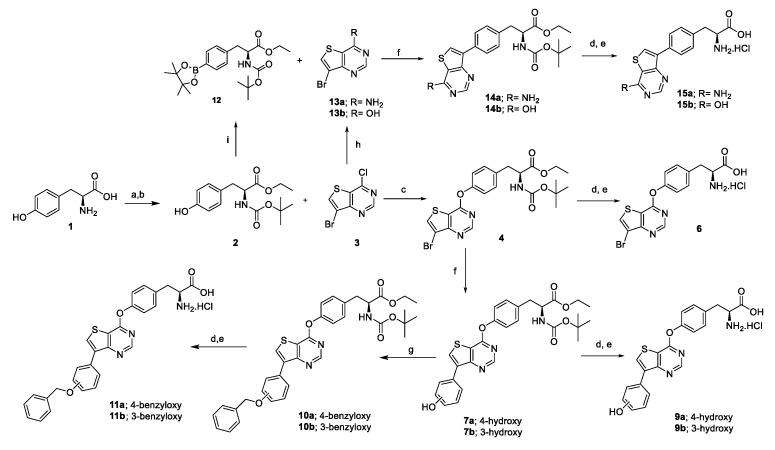
Reagents and conditions: (a) SOCl₂, EtOH, reflux, 2 days, 63%; (b) (Boc)_2_O, TEA, MeOH, 0 °C to rt, 18 h, 96%; (c) Cs_2_CO_3_, ACN, 40 °C, 12 h, 87%; (d) NaOH, THF, H_2_O, rt, 12 h, 93%; (e) 4M HCl in dioxane, EtOAc, rt, 12 h, 50%; (f) **5a**, **b** or **12**, K_2_CO_3_, Pd(PPh_3_)_4_, H_2_O, dioxane, 100 °C, 2 h, 68%; (g) **8**, PPh_3_, DIAD, THF, 0 °C to rt, 18 h, 98%; (h) Ammonia in IPA or aq.NaOH, dioxane, 50%; (i) (1) (CF_3_SO_2_)_2_O, DCM, pyridine, 0 °C-rt, 1 h; (2) Bis(pinacolato) diboron, PdCl_2_(dppf).DCM, KOAc, DMSO, 100 °C, 12 h, 84%.

Tyrosine, which is similar to pCPA, was selected as the starting material because it is suitable for derivatization. The general methods for the synthesis of oxy-phenylalanine derivatives are outlined in Scheme 1. Commercially available L-tyrosine (**1**) was first esterified, and Boc-anhydride was then introduced to provide Boc protection, which yielded compound **2**. Compound **2** was subsequently coupled with 7-bromo-4-chlorothieno[3,2-d] pyrimidine (**3**) to yield compound **4**. Compound **4** was treated with NaOH and subjected to Boc deprotection with HCl (4 M) in 1,4-dioxane to give compound **6**. Compound **4** also underwent Suzuki coupling with 3-hydroxyphenylboronic acid (**5a**) and 4-hydroxyphenylboronic acid (**5b**) in the presence of a palladium catalyst to afford compounds **7a** and **7b**; these compounds were hydrolyzed and underwent Boc deprotection to give compounds **9a** and **9b**. Compounds **7a** and **7b** also underwent the Mitsunobu reaction with benzyl alcohol to afford compounds **10a** and **10b**, which were treated with NaOH and underwent Boc-deprotection with HCl (4 M) in 1,4-dioxane to give compounds **11a** and **11b**. Compounds **15a** and **15b** were also synthesized, as shown in Scheme 1. Thienopyrimidine (**3**) was ammonified to afford compound **13a**, and thienopyrimidine (**3**) was treated with aqueous NaOH to afford compound **13b**. Compounds **12** and **13b** were coupled via Suzuki coupling to afford compounds **14a** and **14b**. Triflic anhydride and bis(pinacolato) diboron were used to prepare compound **12** from compound **2**. Compounds **14a** and **14b** were then hydrolyzed, and subsequent acid deprotection afforded compounds **15a** and **15b**.

**Scheme 2:**
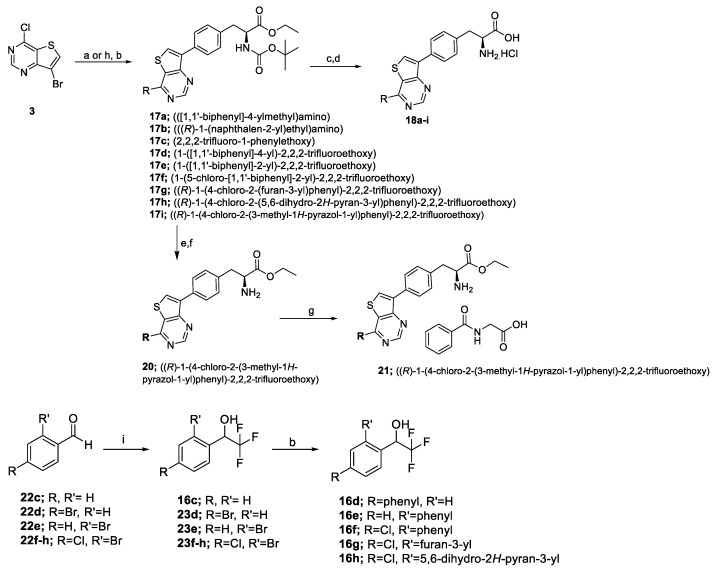
Reagents and conditions: (a) **16c**, **16d**–**i**, NaH, DMF, 0 °C to rt, 96%; (b) **12** or **24a**–**c**, Pd(PPh_3_), K_2_CO_3_, dioxane, H_2_O, 90°C, 3 h, 70%; (c) NaOH, THF, H_2_O, rt; (d) 4M HCl in dioxane, EtOAc, rt, 12 h, 77%; (e) 4M HCl in dioxane, EtOAc, rt, 12 h; (f) aqueous ammonia; (g) hippuric acid, EtOH, reflux, 8 h, 95%; (h) **16a**,**b**, dioxane, Cs_2_CO_3_, rt; (i) CF_3_Si(CH_3_)_3_, CsF, THF, 0 °C-rt, 6 h.

Amino and oxy derivatives were synthesized by substitution at position-4 of the thienopyrimidine ring; the general synthesis is shown in Scheme 2. Commercially available 7-bromo-4-chlorothieno[3,2-d]pyrimidine **3** was condensed with [1,1’-biphenyl]-4-ylmethanamine (**16a**) and (*R*)-1-(naphthalen-2-yl)ethan-1-amine (**16b**), phenyl 2,2,2-trifluoroethan-1-ol (**16c**), and substituted phenyl 2,2,2-trifluoroethan-1-ol (**16d–i**). The condensates subsequently underwent Suzuki coupling with ethyl (*S*)-2-((*tert*-butoxycarbonyl)amino)-3-(4-(4,4,5,5-tetramethyl-1,3,2-dioxaborolan-2-yl)phenyl)propanoate (**12**) to yield compounds **17a–i**, which were subsequently saponified and underwent acidic deprotection to afford several amine hydrochlorides (compounds **18a–i**). Compound **17i** underwent *tert*-butyl deprotection to give compound **19**, which was treated with ammonium hydroxide to give an amine, compound **20**. Hippuric acid was treated with compound **20** to produce a hippurate salt, compound **21**.

The substituted benzaldehydes (**22c**–**h**) were treated with (trifluoromethyl)trime thylsilane and tetrabutylammonium fluoride which yielded compounds **16c** and **23d***–***h**. Compounds **23d***–***h** underwent Suzuki coupling with phenyl boronic acid (**24a**), furan-3-ylboronic acid (**24b**), and 5,6-dihydro-2H-pyran-3-yl)boronic acid (**24c**) which yielded compounds **16d**–**h**. Commercially available (*R*)-1-(4-chloro-2-(3-methyl-1H-pyrazol-1-yl)phenyl)-2,2,2-trifluoroethan-1-ol (**16i**) was used to synthesize compound **18i**.

**Scheme 3:**
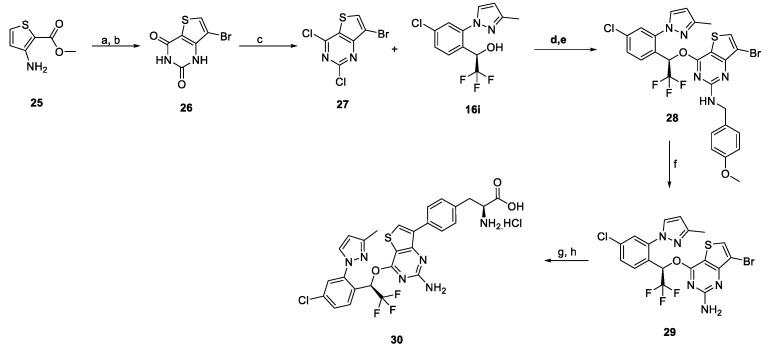
Reagents and conditions: (a) Urea, 190 °C, 51%; (b) Br_2_, AcOH, 81%; (c) N,N-dimethylaniline, POCl_3_, 110 °C, 24 h, 87%; (d) DMF, NaH, 0 °C to rt, 46%; (e) 4-methoxybenzylamine, EtOH, TEA, MW 150 °C, 98%; (f) TFA, 120 °C, 12 h, 97%; (g) **12**, Pd(PPh_3_), K_2_CO_3_, dioxane, H_2_O, 90 °C, 3 h, 86%; (h) (i) NaOH, THF, H2O, rt; (ii) 4M HCl in dioxane, EtOAc, rt, 12 h.

The synthesis of compound **30** is shown in Scheme 3. Compound **25** was cyclized with urea and then brominated to afford compound **26** which was subsequently chlorinated with POCl_3_ to obtain compound **27**. Compound **27** was further treated with compound **16i** and *p*-methoxybenzylamine to afford compound **28**. TFA deprotection afforded compound **29**, which was then hydrolyzed; acidic deprotection of **29** afforded compound **30**. 

### 2.2. Biological Evaluations

The in-house screening revealed that thienopyrimidine tyrosine (derivative **6**) was active in the inhibition of TPH1, with an inhibition of 64% at 100 µM. Consequently, the core structure of thienopyrimidine tyrosine (derivative **6**) was selected as the starting point for the synthesis, evaluating the structure-activity relationships (SAR), and optimization of TPH1 inhibitors. The ability of the synthesized compounds to inhibit TPH1 at 100 µM and 1 µM, respectively, was evaluated. In the evaluation, pCPA and LP533401 (4-[2-amino-6-[2,2,2-trifluoro-1-(3’-fluoro[1,1’-biphenyl]-4-yl)ethoxy]-4-pyrimidinyl]-L-phenylalanine) were used as references. 

The optimized parameters for the oxy-heteroaryl phenylalanine derivatives of compound **6** are summarized in Table 1. Compound **6** exhibited an inhibition that was greater than 60%. When substituents were introduced at the *para* position (compounds **9a** and **11a**), little loss of the inhibitory activity occurred (45% and 58% inhibition at 100 µM, respectively). Compounds **9b** and **11b** (82% and 94% inhibition at 100 µM, respectively) showed better inhibitory activity than compounds **9a** and **11a**, respectively.

**Figure 2:**
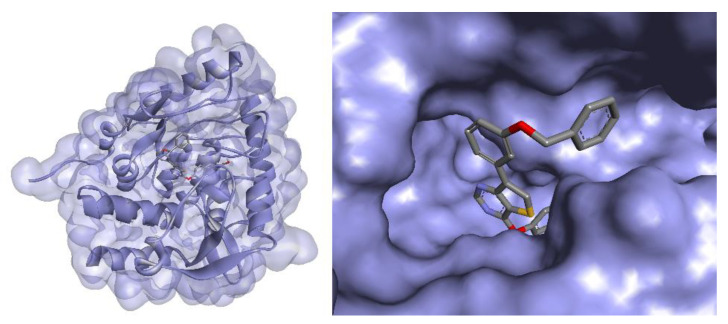
Binding analysis of compound 11b: Predicted binding mode of compound **11b** with TPH1. TPH1 is shown as ribbons, while compound **11b** is represented by sticks. The inset (**left**) shows the binding of compound **11b** to the substrate-binding pocket of TPH1. The zoomed-in view (**right**) highlights the hydrophobic tail of compound **11b** binding at the exterior of the active site of TPH1 with weak interactions. The entrance of the active site of TPH1 is not occupied with compound **11b** which is required for the effective inhibition of TPH1.

Based on these results, computational molecular docking studies were conducted to improve the molecular level understanding of effective binding to the active site; these studies are fundamental for facilitating further structural modification. For the docking studies, the high-resolution structure of human TPH1 (Protein Data Bank access code 3HF8; chain A) was used in AutoDock4.2 [11,12]. The standard protocols and parameters described in our previous work were used to pre-process the structures [13]. The molecular docking results showed that compound **11b** interacted with the catalytic residues of TPH1 (Arg257 and Thr265). In addition, the hydrophobic tail of compound **11b** was bound to the exterior of the active site of TPH1 with weak interactions and unable to block the entrance of the TPH1 active site. Such binding of the hydrophobic tail of compound **11b** is leaving the possibility of substrate binding with the TPH1 in a competitive manner (Figure 2). We hypothesized that the interaction of the hydrophobic tail of synthesized compounds at the entrance of the active site of TPH1 might prevent the substrate access the active site of TPH1 which might lead to better inhibition. Based on these observations and our hypothesis, we first replaced oxy-phenylalanine to produce compounds **15a**, **15b,** and **18a**. Although compounds **15a** and **15b** showed weak activity, biphenyl methyl amine (**18a**) was a promising TPH1 inhibitor with an inhibition of 90% at 100 µM.

Based on these results, we explored a wide range of substituents on position-4 of the thienopyrimidine core to enhance the inhibitory activity; the results are summarized in Table 2. Naphthalene ethane amine (**18b**) showed less inhibition than compound **18a**. Attempts were made to introduce benzyloxy derivatives at position-4 of thienopyrimidine. However, these derivatives were unstable under the acidic conditions used for Boc deprotection, as evidenced by the cleavage of the benzyl moiety to form compound **15b**. To improve the stability of these derivatives, attempts were made to synthesize **18c**, **18d**, and **18e,** which have a CF_3_-substituted benzyloxy moiety. The molecular docking study shows that the CF_3_ group interacted with Leu236 and Phe241 of TPH1. Contrastingly, compound **18c** without a substituent and compound **18d** with a phenyl substituent at the *para* position on the phenyl ring completely lost their activity. However, when the substituent was in the *ortho* position (compound **18e**), the percentage inhibition dramatically improved at a concentration of 1 µM. Although the interactions among compound **18e** and the catalytic and key residues of TPH1 were enhanced, the hydrophobic tail of compound **18e** partially occupied the entrance of the active site of TPH1. This observation suggests that further modifications are required. Halogens are widely used in drug design to improve the drug-target binding affinity and slow drug metabolism; therefore, we introduced chlorine at the *para* position of the benzyloxy moiety. However, the introduction of chlorine in **18e** yielded compound **18f**, which showed a loss in the percentage inhibition. Based on the molecular docking simulation, the *R* isomer of the trifluoroethoxy linker was also introduced as a substituent. This approach resulted in the design and synthesis of compounds **18g**, **18h**, and **18i** with furyl, dihydropyran, and methyl pyrazole substituents. Compared with compound **18g**, compounds **18h** and **18i** showed a higher percentage inhibition, with *IC*_50_ values of 263 nM and 37 nM, respectively.

To optimize the binding of the CF_3_ group and thienopyrimidine ring with TPH1, compound **18i** was synthesized as an *R*-enantiomer with a modified hydrophobic tail, which was capable of effectively inhibiting TPH1. The optimal interactions were observed among the thienopyrimidine ring and phenylalanine moiety of compound **18i** and the aforementioned key and catalytic residues of TPH1.

Therefore, compound **18i** is a more effective inhibitor than compounds **18e** and **18h** because of its increased potency, as evidenced by its *IC*_50_ value of 37 nM. It was further assumed that adding an amine group to the thienopyrimidine ring would likely confer a different potency profile to compound **18i**. However, compound **30** disproved this assumption because it showed reduced potency, as evidenced by the *IC*_50_ value of 208 nM (Table 3). During this evaluation, LP533401 was used as a reference [14]. The prodrugs of compound **18i** were synthesized to improve its oral absorption (Table 3). The ester of compound **18i**, compound **19**, exhibited nanomolar inhibitory activity, with an *IC*_50_ value of 994 nM. In addition, the hippurate salt (**21**) was synthesized as an oral drug for in vivo study.

Based on the in vitro data, compound **18i** was selected for inhibiting the in vitro BBB permeability. Several in vitro methods and computational models have been used in drug discovery to predict the potential of a drug to penetrate the BBB [15,16,17,18,19,20,21]. The parallel artificial membrane permeation assay (PAMPA) was used to evaluate the BBB permeability of the selected compounds. The PAMPA-BBB assay is advantageous because it predicts passive penetration at the blood-brain barrier with high success, high throughput, low cost, and high reproducibility [21].

Table 4 shows that compound **18i** has non-BBB permeable characteristics, with *Pe* ≈ 0.00 × 10^−6^ cm/s (i.e., *Pe* < 2.0), whereas pCPA exhibited a higher *Pe* of 1.85 × 10^−6^ cm/s. Therefore, compound **18i** was chosen as a prototype for further investigation. Compound **18i** showed good liver microsomal stability (99% of the parental microsomes remained after incubation for 30 min). Table 5 shows that compound **18i** did not significantly inhibit any CYP isoforms (1A2, 2C19, 2D6, and 3A4).

Compound **18i** was evaluated for its BBB ratio by iv injection and PK profile in rats as shown in Table 6. The levels of compound **18i** in the plasma (2266 and 1439 ng/mL) indicate that it did not penetrate the BBB. Furthermore, the level of compound **18i** in the plasma was below the quantification limit (7 ng/mL). The PK profiles of **18i** were also evaluated which revealed that compound **18i** had a reasonable AUC of 1.2 μg h/mL; however, **18i** itself could not be absorbed into the body due to its low permeability. Therefore, **19** and **21** were introduced as the ethyl ester and hippurate salt prodrug, respectively, affording improved oral exposure, with 16% oral bioavailability.

**Figure 3:**
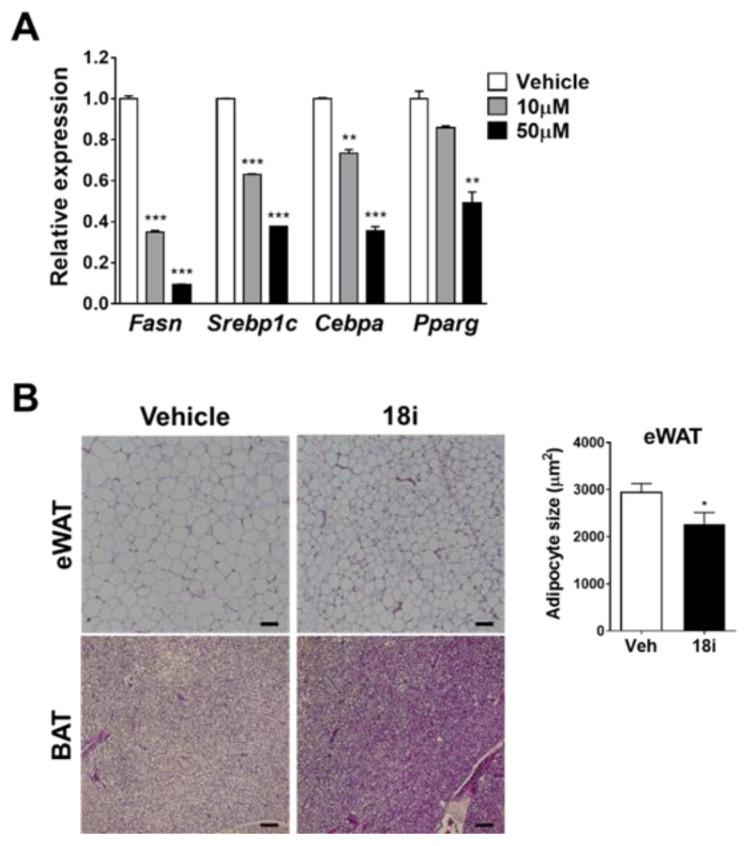
Compound **18i** protects against diet-induced obesity. (**A**) Lipogenic gene expression in 3T3-L1 cells treated with vehicle or **18i** at the concentration of 10 μM or 50 μM. This experiment was repeated as biological triplicates with similar results (**B**) Representative haematoxylin and eosin (H&E) images of epididymal WAT (eWAT) and brown adipose tissue (BAT) from HFD-fed mice treated with vehicle or compound **18i**-treated for 10 days (left, scale bar is 100 μm). Average adipocyte cross-section area of eWAT measured from H&E images (right). * *p* < 0.05, ** *p* < 0.01 and *** *p* < 0.001 vs. vehicle by Student’s *t*-test. Data are presented as mean ± s.e.m.

**Figure 4:**
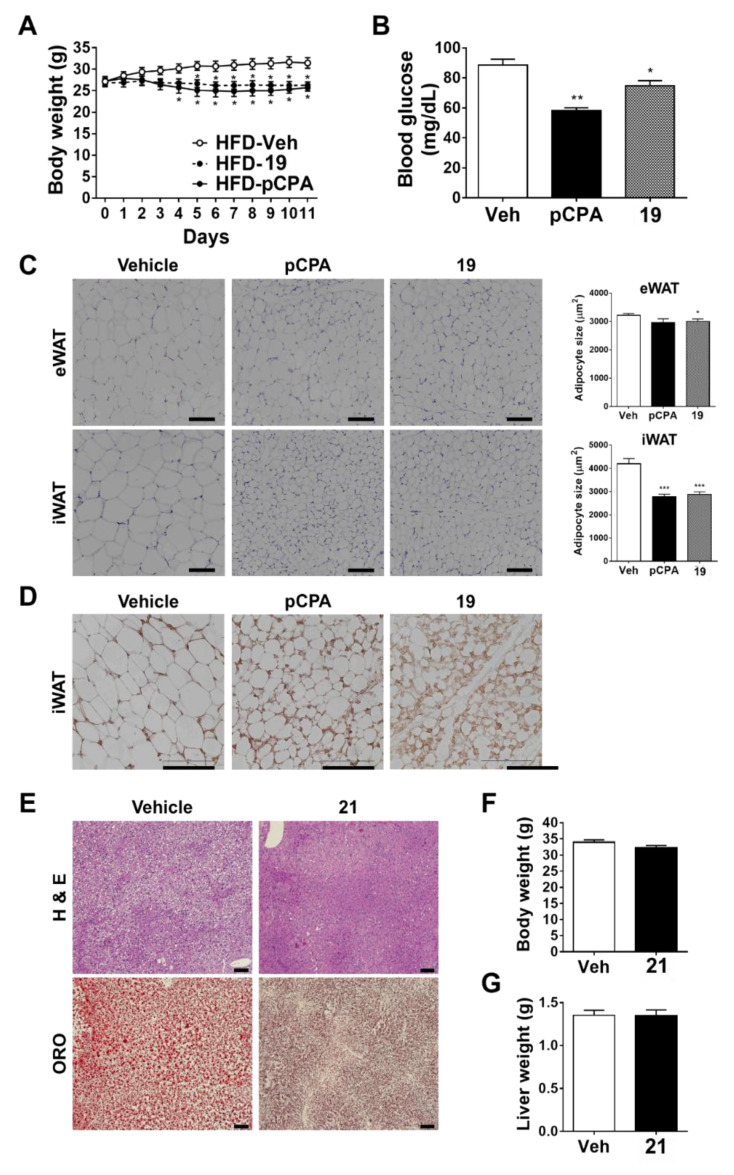
Compounds **19** and **21** show in vivo efficacy for obesity and fatty liver diseases. (**A**) Body weight of HFD-fed mice treated with vehicle (*n* = 6), 300 mg kg^−1^ pCPA (*n* = 3) or 100 mg kg^−1^ **19** (*n* = 4) for 11 days. (**B**) Fasting blood glucose levels in HFD-fed mice after treatment of vehicle (*n* = 6), pCPA- (*n* = 3) or **19** (*n* = 4). (**C**) Representative H&E images of iWAT and eWAT from vehicle-, pCPA- or **19**-treated mice fed a HFD (left, scale bar = 100 μm). The average adipocyte cross-section area of iWAT and eWAT measured from H&E images (right). (**D**) Immunohistochemistry of UCP1 protein in iWAT sections from HFD-fed mice treated with vehicle, pCPA, or compound **19** (scale bar = 100 μm) (**E**) Representative liver sections stained with H&E and Oil-red O (ORO) from HFD-fed mice treated with vehicle or compound **21** (scale bar = 100 μm). (**F**) Body and (**G**) liver weights after 6 weeks of vehicle or compound **21** treatment to HFD-fed mice. * *p* < 0.05, ** *p* < 0.01 and *** *p* < 0.001 vs. vehicle by Student’s *t*-test. Data are presented as mean ± s.e.m.

Recent reports have shown that TPH inhibitors, such as pCPA and LP533401, protect against diet-induced obesity in vivo by reducing body weight gain and lipogenesis in adipose tissue in high-fat diet (HFD) fed mice [7,22]. Based on the pharmacological profiles of newly developed TPH1 inhibitors, we selected compound **18i** and its prodrug compound **19** and **21** for further efficacy tests in HFD-induced obesity mouse models. First, we tested the effects of compound 18i in vitro by treating 3T3-L1 adipocyte differentiation and found decreased expression of key lipogenic genes, Fasn and Srebp1c, along with the adipogenic transcription factors, Pparg and Cebpa (Figure 3A), indicating an anti-adipogenic effect for compound **18i** during adipocyte differentiation. To assess in vivo efficacy, we performed daily intraperitoneal injections of compound **18i** to HFD-fed C57BL6 mice. We found that compound **18i** treatment, as compared to vehicle, resulted in blunted weight gain accompanied by a significant reduction in epididymal white adipose tissue (eWAT) and brown adipose tissue (BAT) adipocyte size as assessed by histological examination (Figure 3B). Similar to the effects of compound **18i**, compound **19** treatment attenuated body weight gain in HFD-fed mice compared to vehicle (Figure 4A). In addition to decreased adiposity, HFD-fed mice treated with compound **18i** had lower fasting blood glucose levels compared to vehicle-treated mice (Figure 4B). Histological analysis of adipose tissues from compound **19**-treated mice showed decreased adipocyte size in both eWAT and inguinal white adipose tissue (iWAT) (Figure 4C). iWAT from compound **19** treated mice had a higher content of mitochondrial uncoupling protein 1 (UCP1), a marker for thermogenic beige adipose tissue, as assessed by immunohistochemistry (Figure 4D). Notably, oral administration of compound **21** to HFD-fed mice dramatically decreased lipid accumulation in liver tissue compared to vehicle-treated mice (Figure 4E), as assessed by histological examination and oil red O staining, without significant changes in body and liver weights (Figure 4F,G). This effect by compound **21** treatment is consistent with our finding that gut tissue-specific deletion of TPH1 in mice leads to a reduction in liver steatosis in HFD-fed mice without affecting body and liver weights (unpublished data). Taken together, these results demonstrate the in vivo efficacy of our newly developed TPH1 inhibitors for ameliorating obesity and fatty liver diseases.

**Figure 5:**
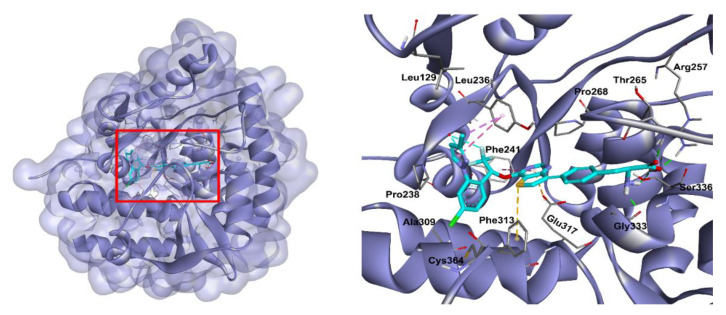
The predicted binding mode of inhibitor **18i** with TPH-1. TPH-1 is shown in ribbon representation with a partially transparent surface while the compound **18i** is shown as sticks. The inset (**left**) shows the optimal binding of compound 18i at the active site of TPH-1 marked with the red box and the zoomed-in view (**right**) highlights the key interactions of hydrogen bonds, pi-sulfur, fluorine interactions, and hydrophobic interactions, which were depicted with green, yellow, cyan, and pink, respectively, according to their interaction type. The alkyl interactions were not shown for ease of presentation and clarity, which are explained in the main text. Figures were prepared with Discovery Studio Visualizer, Dassault Systèmes.

Docking studies revealed that compound **18i** had optimal interactions with the catalytic and other important residues of TPH1, with predicted binding free energy of −14.33 kcal/mol, which effectively inhibited TPH1. The amino group in the phenylalanine moiety of compound **18i** interacted with the catalytic residues, Arg257 and Thr265, and the TPH1 residues, Gly333 and Ser335 (Figure 5). The phenyl ring of the phenylalanine moiety interacted with His272 and Phe318 of TPH1. The phenyl ring of the phenylalanine moiety and the thienopyrimidine ring were optimally oriented because of Pro268 of TPH1, which facilitated the interaction of the thienopyrimidine ring with the key residues, Phe241 and Glu317. Additional interactions were observed between the sulfur of the thienopyrimidine ring and the Phe313 residue of TPH1. Modification of the hydrophobic tail of compound **18i** improved its interactions, thereby blocking the entrance of the active site of TPH1. To summarize, compound **18i** adopts a compact conformation that is expected to inhibit the binding of substrate cofactor and interferes with the binding of iron with TPH1, thereby leading to the effective inhibition of TPH1. 

## 3. Experimental Section

### 3.1. Chemistry

All reported yields are isolated yields after column chromatography or crystallization. All solvents and chemicals were used as purchased without further purification. ^1^H NMR spectra and ^13^C spectra were recorded on a JEOL JNM-ECS400 spectrometer at 400 MHz for ^1^H NMR and 100 MHz for ^13^C NMR, respectively. The chemical shift (δ) is expressed in ppm relative to tetramethylsilane (TMS) as an internal standard, and CDCl3, DMSO-d6, and CD3ODwere used as solvents. The multiplicity of peaks is expressed as s (singlet), d (doublet), t (triplet), q (quartet), dd (doublet of doublets), td (triplet of doublets), qd (quartet of doublets), dt (doublet of triplets), and m (multiplet). Melting points were determined on a Melting Point M-560, purchased from Buchi. Fast atom bombardment-high-resolution mass spectrometry (FAB-HRMS) data were obtained by a JMS 700 (JEOL, Japan). The purity of all tested compounds was ≥ 95%, as estimated by high performance liquid chromatography (HPLC) analysis. Samples were analyzed on a Waters Agilent HPLC system equipped with a PDA detector and a Waters SB-C18 column (1.8 μm, 2.1 × 50 mm^2^). The mobile phase was used with buffer A (ultrapure H2O containing 0.1% TFA) and buffer B (chromatographic-grade CH3CN). The flow rate was 0.5 mL/min.

### 3.2. Synthesis of (S)-2-amino-3-(4-(4-((R)-1-(4-chloro-2-(3-methyl-1H-pyrazol-1-yl)phenyl)-2,2,2-trifluoroethoxy)thieno[3,2-d]pyrimidin-7-yl)phenyl)propanoic acid hydrochloride (**18i**)

#### 3.2.1. Ethyl (S)-2-((tert-butoxycarbonyl)amino)-3-(4-(4-((R)-1-(4-chloro-2-(3-methyl-1H-pyrazol-1-yl)phenyl)-2,2,2-trifluoroethoxy)thieno[3,2-d]pyrimidin-7-yl)phenyl)propanoate (**17i**)

(R)-1-(4-chloro-2-(3-methyl-1H-pyrazol-1-yl)phenyl)-2,2,2-trifluoroethan-1-ol **16i** (900 mg, 3.096 mmol) was dissolved in 4 mL of N,N-dimethylformamide and cooled to 0 °C. Sodium hydride 60% in oil (185.7 mg, 4.644 mmol) was added to the mixture and stirred for 60 min. 7-bromo-4-chloro-thieno[3,2-d]pyrimidine **3** (811.18 mg, 3.25 mmol) was added to the mixture and stirred at room temperature for 12 hr. The resulting mixture was quenched in aqueous ammonium chloride and extracted twice with ethyl acetate. The organic layer was washed with water and brine, dried over anhydrous sodium sulfate, and concentrated under a reduced pressure to obtain a residue. The resulting residue was purified by column chromatography to obtain of the title compound (R)-7-bromo-4-(1-(4-chloro-2-(3-methyl-1H-pyrazol-1-yl)phenyl)-2,2,2-trifluoroethoxy)thieno[3,2-d]pyrimidine (1.5 g, 96%).

(R)-7-bromo-4-(1-(4-chloro-2-(3-methyl-1H-pyrazol-1-yl)phenyl)-2,2,2-trifluoroethoxy) thieno [3,2-d]pyrimidine (1.5 g, 2.978 mmol) was added to 1,4-dioxane (25 mL), and ethyl (S)-2-((tert-butoxycarbonyl)amino)-3-(4-(4,4,5,5-tetramethyl-1,3,2-dioxaborolan-2-yl)phenyl) propanoate **12** (1.37 g, 3.26 mmol)), tetrakis(triphenylphosphine)palladium(0) (172.06 mg, 0.149 mmol), potassium carbonate (823.11 mg, 5.96 mmol), and water (8 mL) were sequentially added thereto while stirring. The reaction mixture was heated to 90 °C, and stirred at 90 °C for 3 h. After completion of the reaction using brine, the reaction mixture was extracted twice with ethyl acetate. The collected organic layer was dried over anhydrous sodium sulfate and concentrated under reduced pressure to obtain a foamy residue which was purified by column chromatography to obtain the title compound ethyl (S)-2-((tert-butoxycarbonyl)amino)-3-(4-(4-((R)-1-(4-chloro-2-(3-methyl-1H-pyrazol-1-yl)phenyl)-2,2,2-trifluoroethoxy)thieno[3,2-d]pyrimidin-7-yl)phenyl)propanoate **17i** (1.5 g, 70%).^1^H NMR (400 MHz, DMSO-d_6_): δ 8.70 (s, 1H), 8.67 (s, 1H), 8.24 (d, J = 2.44 Hz, 1H), 7.99 (d, J = 8.24 Hz, 2H), 7.88–7.78 (m, 2H), 7.70 (d, J = 2.14 Hz, 1H), 7.56 (dd, J = 8.54, 2.14 Hz, 1H), 7.34 (d, J = 8.24 Hz, 2H), 6.40 (d, J = 2.44 Hz, 1H), 4.32–4.25 (m, 1H), 4.11 (q, J = 5.19 Hz, 2H), 3.22–3.06 (m, 2H), 2.27 (s, 3H), 1.33 (s, 9H), 1.09 (t, J = 7.02 Hz, 3H).

#### 3.2.2. (S)-2-Amino-3-(4-(4-((R)-1-(4-chloro-2-(3-methyl-1H-pyrazol-1-yl)phenyl)-2,2,2-trifluoro ethoxy)thieno[3,2-d]pyrimidin-7-yl)phenyl)propanoic acid hydrochloride (**18i**)

NaOH (50.27 mg, 1.26 mmol) was added to a solution of ethyl (S)-2-((tert-butoxycarbonyl)amino)-3-(4-(4-((R)-1-(4-chloro-2-(3-methyl-1H-pyrazol-1-yl)phenyl)-2,2,2-trifluoroethoxy)thieno[3,2-d]pyrimidin-7-yl)phenyl)propanoate **17i** (180 mg, 0.251 mmol) in THF/water (50 mL, 3:1). The reaction mixture was stirred at ambient temperature for 24 h. The THF was removed in vacuo and the resulting solution was acidified with 1N hydrochloric acid to pH 4. More water was added (50 mL) and the aqueous solution was extracted with EtOAC (3 × 50 mL). The combined organic layer was washed with brine, dried over sodium sulfate, and concentrated. A crude product was purified by column chromatography to afford the title compound (S)-2-((tert-butoxycarbonyl)amino)-3-(4-(4-((R)-1-(4-chloro-2-(3-methyl-1H-pyrazol-1-yl)phenyl)-2,2,2-trifluoroethoxy) thieno [3,2-d]pyrimidin-7-yl)phenyl)propanoic acid.

Hydrogen chloride 4.0 M solution in 1,4 dioxane (5 mL) was added to a mixture of (S)-2-((tert-butoxycarbonyl)amino)-3-(4-(4-((R)-1-(4-chloro-2-(3-methyl-1H-pyrazol-1-yl)phenyl)-2,2,2-trifluoroethoxy)thieno[3,2-d]pyrimidin-7-yl)phenyl) propanoic acid in ethyl acetate (10 mL) and the mixture was stirred for 12 h. The mixture was concentrated to minimum volume and the residue was collected by filtration to give (S)-2-amino-3-(4-(4-((R)-1-(4-chloro-2-(3-methyl-1H-pyrazol-1-yl)phenyl)-2,2,2-trifluoro ethoxy)thieno[3,2-d]pyrimidin-7-yl)phenyl)propanoic acid hydrochloride **18i** (121 mg, 77%) (over two steps) as an off white solid. ^1^H NMR (400 MHz, DMSO-d_6_): δ 13.83 (s,1H), 8.70 (d, J = 1.22 Hz, 1H), 8.66 (d, J = 1.53 Hz, 1H), 8.37 (bs, 3H), 8.24 (s, 1H), 7.98 (d, J = 7.32 Hz, 2H), 7.88–7.77 (m, 1H), 7.72–7.68 (m, 1H), 7.56 (dt, J = 8.54,1.22 Hz, 1H), 7.37 (d, J = 7.63 Hz, 2H), 6.43–6.39 (m, 1H), 4.24–4.15 (m, 1H), 3.14 (d, J = 6.10 Hz, 2H), 2.27 (s, 3H); ^13^C NMR (100 MHz, DMSO-d_6_): δ 170.34, 157.49, 154.71, 149.94, 146.62, 139.73, 136.26, 134.47, 134.03, 132.74, 132.59, 130.95, 130.74, 129.65, 129.47, 128.17, 127.95, 124.97, 124.40, 107.27, 53.10, 35.46, 13.38; HRMS (FAB) *m*/*z* calcd for C_27_H_21_ClF_3_N_5_O_3_S [M + H]^+^ 587.1006, found 588.1089; LC-MS (*m*/*z*): 588.2 [M + H]; HPLC purity 99.12%; m.p. 215–217 °C.

### 3.3. Synthesis of ethyl (S)-2-amino-3-(4-(4-((R)-1-(4-chloro-2-(3-methyl-1H-pyrazol-1-yl)phenyl)-2,2,2-trifluoroethoxy)thieno[3,2-d]pyrimidin-7-yl)phenyl)propanoate hydrochloride (**19**)

Hydrogen chloride 4.0 M solution in 1,4 dioxane (5 mL) was added to a mixture of ethyl (S)-2-((tert-butoxycarbonyl)amino)-3-(4-(4-((R)-1-(4-chloro-2-(3-methyl-1H-pyrazol-1-yl)phenyl)-2,2,2-trifluoroethoxy)thieno[3,2-d]pyrimidin-7-yl)phenyl)propanoate (500 mg, 0.698 mmol) in ethyl acetate (25 mL) and the mixture was stirred for 12 h. The mixture was concentrated and filtered to give title compound ethyl (S)-2-amino-3-(4-(4-((R)-1-(4-chloro-2-(3-methyl-1H-pyrazol-1-yl)phenyl)-2,2,2-trifluoroethoxy)thieno[3,2-d]pyrimidin-7-yl)phenyl)propanoate hydrochloride **19** (405 mg, 88%) as an off white solid. ^1^H NMR (400 MHz, DMSO-d_6_): δ 8.75 (d, J = 3.05 Hz, 1H), 8.62 (d, J = 3.05 Hz, 1H), 8.26 (t, J = 2.44 Hz, 1H), 7.96 (dd, J = 7.93, 2.44 Hz, 2H), 7.89–7.82 (m, 2H), 7.72 (t, J = 2.44 Hz, 1H), 7.59 (dt, J = 8.85, 2.44 Hz, 1H), 7.32 (dd, J = 8.24, 2.14 Hz, 2H), 6.44 (t, J = 2.44 Hz, 1H), 4.02 (q, J = 7.02 Hz, 2H), 3.63–3.58 (m, 1H), 2.95–2.77 (m, 2H), 2.30 (s, 3H), 1.13 (t, J = 7.02 Hz, 3H); ^13^C NMR (100 MHz, DMSO-d_6_): δ 168.95, 161.37, 159.95, 135.74, 150.48, 140.66, 135.51, 135.18, 134.65, 133.27, 132.71, 131.92, 130.16, 129.75, 128.13, 128.08, 124.63, 123.83, 117.82, 107.82, 61.71, 53.08, 35.66, 13.83, 13.15; HRMS (FAB) *m*/*z* calcd for C_29_H_25_ClF_3_N_5_O_3_S [M + H]^+^ 615.1319, found 616.1399; LC-MS (*m*/*z*): 616.05 [M + H]; HPLC purity 98.8%; m.p. 102–104 °C.

### 3.4. Synthesis of ethyl (S)-2-amino-3-(4-(4-((R)-1-(4-chloro-2-(3-methyl-1H-pyrazol-1-yl)phenyl)-2,2,2-trifluoroethoxy)thieno[3,2-d]pyrimidin-7-yl)phenyl)propanoate (**20**)

Hydrogen chloride 4.0 M solution in 1,4 dioxane ( 10 mL) was added to a mixture of ethyl (S)-2-((tert-butoxycarbonyl)amino)-3-(4-(4-((R)-1-(4-chloro-2-(3-methyl-1H-pyrazol-1-yl)phenyl)-2,2,2-trifluoroethoxy)thieno[3,2-d]pyrimidin-7-yl)phenyl)propanoate (1.2 g, 1.676 mmol) in ethyl acetate (20 mL) and the mixture was stirred for 12 h. The mixture was concentrated and the residue was dissolved in water. The pH was adjusted to 8 by aqueous ammonia and extracted with ethyl acetate. The combined organic layer was dried over anhydrous sodium sulfate and concentrated in vacuo. The residue was purified by silica gel column chromatography to give title compound ethyl (S)-2-amino-3-(4-(4-((R)-1-(4-chloro-2-(3-methyl-1H-pyrazol-1-yl)phenyl)-2,2,2-trifluoroethoxy)thieno[3,2-d]pyrimidin-7-yl)phenyl)propanoate **20** (910 mg, 88%) as an off white solid. ^1^H NMR (400 MHz, DMSO-d_6_): δ 8.72 (d, J = 3.05 Hz, 1H), 8.64 (d, J = 3.05 Hz, 1H), 8.26 (t, J = 2.44 Hz, 1H), 7.94 (dd, J = 7.93, 2.44 Hz, 2H), 7.89–7.80 (m, 2H), 7.72 (t, J = 2.44 Hz, 1H), 7.59 (dt, J = 8.85, 2.44 Hz, 1H), 7.30 (dd, J = 8.24, 2.14 Hz, 2H), 6.44 (t, J = 2.44 Hz, 1H), 4.04 (q, J = 7.02 Hz, 2H), 3.62–3.55 (m, 1H), 2.96–2.78 (m, 2H), 2.30 (s, 3H), 1.12 (t, J = 7.02 Hz, 3H); ^13^C NMR (100 MHz, DMSO-d_6_): δ 173.91, 161.34, 159.98, 153.65, 150.46, 140.67, 135.48, 132.68, 130.13, 129.60, 129.50, 127.95, 127.81, 124.63, 123.85, 107.79, 60.25, 55.23, 36.70, 14.01, 13.48; HRMS (FAB) *m*/*z* calcd for C_29_H_25_ClF_3_N_5_O_3_S [M + H]^+^ 615.1319, found 616.14; LC-MS (*m*/*z*): 616.0 [M + H]; HPLC purity 99.24%; m.p. 180–182 °C.

### 3.5. Synthesis of ethyl (S)-2-amino-3-(4-(4-((R)-1-(4-chloro-2-(3-methyl-1H-pyrazol-1-yl)phenyl)-2,2,2-trifluoroethoxy)thieno[3,2-d]pyrimidin-7-yl)phenyl)propanoate hippurate (**21**)

To a solution of ethyl (S)-2-amino-3-(4-(4-((R)-1-(4-chloro-2-(3-methyl-1H-pyrazol-1-yl)phenyl)-2,2,2-trifluoroethoxy)thieno[3,2-d]pyrimidin-7-yl)phenyl)propanoate (330 mg, 0.536 mmol) in ethanol (50 mL) was added hippuric acid (95.98 mg, 0.536 mmol). The reaction mixture was stirred at reflux for 12 h and cooled to room temperature. The reaction mixture was concentrated under reduced pressure to obtain a bubble residue. The resulting residue was dissolved in methyl tert-butyl ether (25 mL) and added n-Hexane to form a solid which filtered to give ethyl (S)-2-amino-3-(4-(4-((R)-1-(4-chloro-2-(3-methyl-1H-pyrazol-1-yl)phenyl)-2,2,2-trifluoroethoxy)thieno[3,2-d]pyrimidin-7-yl)phenyl)propanoate hippurate **21** as a white solid. ^1^H NMR (400 MHz, DMSO-d_6_): δ 8.77 (t, J = 5.49 Hz, 1H), 8.72 (s, 1H), 8.65 (s, 1H), 8.27 (d, J = 2.44 Hz, 1H), 7.95 (d, J = 7.93 Hz, 2H), 7.89–7.80 (m, 5H), 7.72 (d, J = 2.14 Hz, 1H), 7.59 (dd, J = 8.54, 2.14 Hz, 1H), 7.57–7.44 (m, 4H), 7.31 (d, J = 8.24 Hz, 2H), 6.44 (d, J = 2.14 Hz, 1H), 4.05 (q, J = 7.02 Hz, 2H), 3.90 (d, J = 5.80 Hz, 2H), 3.66–3.58 (m, 1H), 2.96–2.80 (m, 2H), 2.31 (s, 3H), 1.09 (t, 7.02 Hz, 3H); ^13^C NMR (100 MHz, DMSO-d_6_): δ 171.44, 166.34, 161.36, 160.00, 153.67, 150.48, 140.69. 135.57, 135.51, 133.97, 132.81, 132.71, 131.34, 131.22, 130.16, 129.64, 129.52, 128.34, 128.00, 127.83, 127.22, 124.66, 123.87, 117.78, 107.82, 60.26, 55.30, 41.50, 36.66, 14.03, 13.50; HRMS (FAB) *m*/*z* calcd for C_29_H_25_ClF_3_N_5_O_3_S [M + H]^+^ 615.1319, found 615.7; LC-MS (*m*/*z*): 616.2 [M + H]^+^; HPLC purity 98.79%; m.p. 87–89 °C.

### 3.6. Biology

#### 3.6.1. Microsomal Activity

For the liver microsomal activity, test compounds were incubated with rat and mouse’s liver microsomes, respectively, at a final concentration of 1 µM, in the presence of an NADPH regeneration system in 0.1 M potassium phosphate buffer (pH 7.4) for 30 min h at 37 °C in shaking incubator. The microsomal reaction was terminated by adding four volumes of ice-cold acetonitrile and then centrifuged at 4000 rpm for 20 min. The supernatant was collected and analyzed by a LC-MS/MS system. The percentage of the parent compound remaining was calculated by comparing peak areas.

#### 3.6.2. CYP Inhibition Assay

Five major screening systems: P450-Glo CYP1A2 Screening System (Catalog# V9770), P450-Glo CYP2C9 Screening System (Catalog# V9790), P450-Glo CYP2C19 (Catalog# V9880), P450-Glo CYP 2D6 (Catalog# V9890), and P450-Glo CYP3A4 (Catalog# V9920) were attained from Promega Corp. (Madison, WI). α-naphthoflavone, sulfaphenazole, amitriptyline, quinidine, and ketoconazole were identified as positive control inhibitors for CYP1A2, CYP2C9, CYP2C19, CYP2D6, and CYP3A4, respectively. The CYP enzyme and substrate were combined in a potassium phosphate (KPO4) buffer and the reaction is initiated by adding an NADPH regenerating system. The volume of the mixture (12.5 μL in a 96-well plate) was combined with an equal volume of test compound solution and positive control inhibitor (12.5 μL added to make the volume 25 μL). The reaction of the NADPH regeneration system was initiated by adding a 2× concentration of NADPH solution. Then an equal volume of luciferin detection reagent was added. The plates were incubated at room temperature for 20 min and the luminescence signal was measured by a microplate reader device.

#### 3.6.3. The hERG Inhibition Activity

The inhibition of binding affinity by small molecules was measured using the Predictor™ hERG FP kit (Thermo Fisher Scientific, Inc., Rockford, IL, USA). The procedure was carried out in accordance with the manufacturer’s instructions. Briefly, tracer (Predictor™ hERG tracer red) was prepared with dilution in the binding buffer. The binding assay was conducted in 384 well black flat-bottom microplates (Corning Life Sciences, Lowell, MA, USA) after the serial addition of 5 μL of a 4 nM tracer, 5 μL of test compounds, and a 10 μL membrane fraction containing hERG channel protein. The mixtures were incubated at room temperature for 2 h. The fluorescence polarization (FP) was measured with an excitation filter of 530 nm and an emission filter of 585 nm using a microplate reader (Infinite M1000PRO; Tecan, Mannedorf, Switzerland).

#### 3.6.4. Blood Brain Barriers PK

The biodistribution study in plasma and brain tissue was performed using eight-week-old male SD rats (Orient Bio Inc., Seongnam, Korea). Dosing vehicles were composed of DMSO, PEG400, and deionized water (5:40:55, *v*/*v*/*v*). **18i** compound was administrated at a dose of 5 mg/kg via intravenous (I.V.). Plasma from the inferior vena cava and whole brain tissue were collected at 0.5 and 3 h after drug administration. Brain tissues were rinsed with saline and four volumes of acetronitrile containing disopyramide were added as an internal standard for LC-MS/MS analysis. The brain samples were homogenized using a sonicator (IKA Labortechnik T10 basic ULTRA-TURRAX, Staufen, Germany) for 10 s on the ice. The plasma samples were added to nine volumes of acetonitrile and vortexed for 5 min. The samples were centrifuged (15,000 RPM, 4 °C, 10 min). The supernatant was collected and analyzed by an LC-MS/MS system.

#### 3.6.5. Parallel Artificial Membrane Permeability Assay (PAMPA)

PAMPA was conducted with a STIRWELLTM PAMPA sandwich (pION, Inc., Billerica, MA, USA) consisting of a 96-well filter plate (0.45 μm PVDF membrane) and a 96-well polystyrene donor plate. Test compounds were prepared in 10 mM stock solution with DMSO and diluted to a working solution of 50 μM concentration using a phosphate buffered saline (PBS) buffer. To form lipid membranes in the acceptor plate, 5 μL GIT-0 lipid (pION, Inc., Billerica, MA, USA) was dropped and semi-dried on the bottom side of the 0.45 μm PVDF membrane. The donor plate was prepared by the addition of 200 μL of working solution. The acceptor plate coated with lipid membrane was then overlayed on the donor plate without an air bubble. Continuously, 200 μL of acceptor sink buffer (pION, Inc., Billerica, MA, USA) was dispensed at the inner side of the acceptor plate. The combined cassette plate was incubated for 4 h at room temperature in Gut-BoxTM (pION, Inc., Billerica, MA, USA). To acquire the peak area of range from 230 to 500 nm, absorbance scans of PBS, acceptor, donor, and working solution were carried out with an Epoch ELISA reader (BioTek Instruments Inc., Winooski, VT, USA). The PAMPA Explorer Command Software (pION, Inc., Billerica, MA, USA) was used for the analysis of the permeability of compounds.

#### 3.6.6. Pharmacokinetic Study

The pharmacokinetic study of **18i** was performed using eight-week-old male SD rats (Orient Bio Inc., Seongnam, Korea). **18i** and its prodrug **21** (5 mg/kg, 2 mL/kg) were administered via intravenous and oral routes. Blood samples were collected at 0.033, 0.16, 0.5, 1, 2, 4, 6, 8, and 24 h for intravenous injection at 0.15, 0.5, 1, 2, 4, 6, 8, and 24 h for oral dosing after drug administration, and then immediately centrifuged at 10,000× *g* for 3 min. The plasma concentrations of **18i** were determined by LC-MS/MS. The plasma concentration-time profiles and pharmacokientic parameters were analyzed by a non-compartmental method using the nonlinear least-squares regression program WinNonlin 5.3 (Pharsight, Mountain View, CA, USA).

#### 3.6.7. LC-MS/MS Analysis

The LC-MS/MS analysis was performed on an Agilent 1200 series (Agilent Technologies, Santa Clara, CA, USA) with an API 4000 linear ion trap triple quadruple mass spectrometer (AB Sciex, CA, USA). The chromatographic separation of the drug was carried out using a kinetex column (100 × 2.1 mm, 3 μm, Phenomenex, CA USA) with a SecurityGuard C18 guard column (4 mm × 2.0 mm i.d., Phenomenex). The flow rate was 300 µL/min with a mobile phase consisting of 0.1% formic acid in water (eluent A) and 0.1% formic acid in acetonitrile (eluent B) by linear gradient condition as follows: starting with 0 to 0.3 min, B: 5%; 0.3 to 2.5 min, B: 5 → 95%; 2.5 to 2.9 min, B: 95%; 2.9 to 3.0 min, B: 95 → 5%, and then re-equilibrated to initial conditions until 3 min. The total run time was 6 min and the injection volume of 5 μL.

#### 3.6.8. In Vitro Screening Test

In vitro screening was performed as described previously [PMID: 33417443]. Briefly, TPH1 enzyme activity was measured with a commercially available kit (BPS Bioscience). Compounds were dissolved in DMSO (Sigma). Then, 10 μL of the compound was dispensed into a 96-well microplate, and 40 μL of the TPH1 enzyme solution (320 ng enzyme/reaction) was added. Then, 50 μL of the TPH1 reaction solution was added, and the microplate was sealed with aluminum foil. The microplate was immediately cooled to 4 °C, gently shaken, and incubated for 4 h. After a defined time, 10 μL of the TPH1 quench solution was added. Then, the fluorescence was measured with a Flexstation3 microplate reader at 300 nm for excitation and 360 nm for emission. All experiments were performed in triplicate.

#### 3.6.9. In Vitro Efficacy Test

3T3-L1 cells (American Type Culture Collection, Manassas, VA, USA) were cultured in DMEM supplemented with 10% FCS and 100 mg mL^−1^ P/S in a humidified chamber under 5% atmospheric CO_2_ at 37 °C. Two days after reaching confluence, cells were further cultured in adipocyte differentiation medium (DMEM/10% FBS, P/S, 0.5 mM IBMX, 1 μg mL^−1^ insulin, and 1 μM dexamethasone) (day 0). After 2 days, the medium was changed to adipocyte maintenance medium (DMEM/10% FBS, 1 μg mL^−1^ insulin, P/S) (day 2), and the media was changed every 2 days from day 4 to 8. Cells were treated with compound **6** at 10 or 50 μM concentrations throughout adipocyte differentiation (day 0 thru day 8). On day 8, differentiated 3T3-L1 preadipocytes were harvested for analysis of gene expression.

#### 3.6.10. In Vivo Efficacy Test

All animal study protocols were approved by the Institutional Animal Care and Use Committee at the Korea Advanced Institute of Science and Technology. Male C57BL/6J mice were purchased from SLC (Shizuoka, Japan) and acclimatized for a week. Mice were housed in a humidity and temperature-controlled environment under a 12 h light-dark cycle with ad libitum access to water and food. After acclimatization, male mice were fed a high-fat diet (HFD, 60% fat calories, Research diet; New Brunswick, NJ) and received daily treatment with TPH1 inhibitors. Intraperitoneal administration of PBS or 300 mg kg^−1^ pCPA (Sigma, St. Louis, MO, USA) was performed concomitant to the start of HFD feeding. Compound **18i** was dissolved in 5% dimethyl sulfoxide (DMSO) (Sigma, St. Louis, MO, USA) and 25% β-cyclodextrin (Sigma, St. Louis, MO, USA). Vehicle or compound **18i** (100 mg kg^−1^) was injected intraperitoneally in mice for 10 days after two weeks of HFD feeding. Both compounds **19** and **21** were prepared in 10% DMSO and 10% Kolliphor EL (Sigma, St. Louis, MO, USA). Vehicle and 100 mg kg^−1^ of compound **19** or compound **21** were administered daily by intraperitoneal injection or oral feeding, respectively, concomitant to the initiation of HFD feeding. For analyzing blood glucose concentration, glucose in blood collected from the tail vein was measured after fasting mice for 16 h using a Gluco Dr. Top glucometer (Allemedicus, Anyang, Korea). Body weight was measured daily and mice were sacrificed for harvesting adipose tissue and liver.

#### 3.6.11. Histological Analysis and Immunohistochemistry

Tissue samples were fixed in 10% formalin, embedded in paraffin, and cut into 5 μm thick sections, followed by deparaffinization and rehydration for H&E staining and UCP1 immunohistochemistry. After heat-induced antigen retrieval with a citrate buffer (pH 6.0) for 10 min at 95°C, tissue sections were incubated with BLOXALL Blocking solution (Vector Laboratories, Burlingame, CA, USA) to block endogenous peroxidase, followed by incubation with 2% normal goat serum in phosphate-buffered saline (PBS) for 30 min at room temperature to block nonspecific binding. Sections were incubated overnight with the anti-UCP1 primary antibody diluted 1:200 in 2% normal goat serum in PBS (Abcam, Cambridge, UK) at 4 °C, followed by 30 min incubation with biotinylated anti-rabbit IgG secondary antibody diluted 1:400 in PBS for 30 min at room temperature. After incubation with Vectastain ABC-AP reagent (Vector Laboratories) for 30 min, peroxidase activity was visualized with 3,3′-diaminobenzidine substrates (Vector Laboratories). For measuring average adipocyte size, we analyzed three random fields of H&E stained micrographs per adipose tissue sample from two representative mice of each group using AdipoCount software [23].

#### 3.6.12. Liver Oil-Red O Staining

Fresh liver tissues were flash frozen in O.C.T. compound (Sakura Finetek, Torrance, CA, USA) and 10 μm cryostat sections were collected on glass slides. Sections were allowed to air-dry for 1 h, rinsed with PBS for 5 min, and washed with distilled water for 5 min. After washing, sections were placed in 30% isopropanol for 5 min, 60% isopropanol for 5 min twice, and then stained in Oil Red O solution (0.5% Oil red O (Sigma, St. Louis, MO, USA) dissolved in 60% isopropanol) for 15 min. The stained sections were rinsed twice with distilled water, mounted, coverslipped, and documented using bright field microscopy.

#### 3.6.13. RNA Isolation and Real-Time Quantitative PCR (RT-qPCR)

Total RNA was extracted from 3T3-L1 cells using Trizol reagent (Invitrogen, Carlsbad, CA, USA), purified using a RNeasy Lipid Tissue Mini kit (Qiagen, Hilden, Germany), and an aliquot (2 µg) of total RNA was reversed transcribed using the RevertAidTM First Strand cDNA Synthesis Kit (Thermo Fisher Scientific, Waltham, MA, USA) according to the manufacturer’s instructions. Real-time PCR was carried out using SYBR Green (Power SYBR Green PCR Master Mix, Thermo Fisher Scientific) with a ViiA^™^ 7 Real-Time PCR system (Applied Biosystems, Waltham, MA, USA). Mouse *Rplp0* gene expression was used as the internal control to normalize gene expression values. Primer sequence information for RT-qPCR is shown in Supplementary Materials.

### 3.7. Docking Studies

The experimentally determined structure of TPH1 was downloaded from Protein Data Bank. The choice of three-dimensional structure [PDB access code 3HF8] is based on its high resolution, extensive literature, and our experience with the previous studies. Chain A was retrieved and preprocessed in a Discovery Studio Visualizer with a standard protocol to add missing atoms and residues. The structures of the synthesized compounds were manually drawn in ChemDraw and their 3D conformations were obtained from the Chem3D with the energy minimization protocol. The 3D conformations of TPH1 and synthesized compounds were preprocessed into ‘pdbqt’ files with AutoDockTools [11]. The docking studies were carried out with Lamarckian genetic algorithm implemented in AutoDock4.2 for 200 runs and 270,000 grid points of 0.325 grid spacing that covered the entire active site of TPH1 [11]. The detailed parameterization was described in our previous work [13]. The intermolecular interactions between the synthesized compounds and TPH1 were investigated with Discovery Studio Visualizer.

## 4. Conclusions

A series of oxyphenylalanine and heterocyclic phenylalanine derivatives were identified as TPH1 inhibitors. Among these derivatives, fused heterocyclic derivatives were identified as potent TPH1 inhibitors. Compound **18i** with an *IC*_50_ value of 37 nM was the most active compound in vitro. Compound **18i** showed good liver microsomal stability and did not significantly inhibit CYP and Herg. As a TPH1 inhibitor, this compound was able to interact with the peripheral system without penetrating the BBB. Compound **18i** and its prodrugs, compounds **19** and **21**, reduced body weight gain and decreased in vivo fat accumulation in mammals. Consequently, this compound as a therapeutic agent is a useful non-BBB permeable TPH1 inhibitor that can act on the peripheral system while preventing obesity and fatty liver disease.

## Figures and Tables

**Table 1:** In vitro test of thienopyrimidine derivatives.

Compound	Structure	TPH1 Inhibition (% Inhibition at 100 µM)
**pCPA**	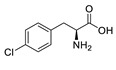	25%
**6**	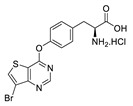	64%
**9a**	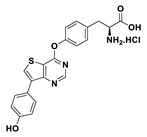	45%
**9b**	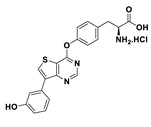	82%
**11a**	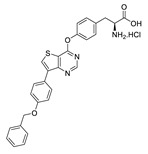	58%
**11b**	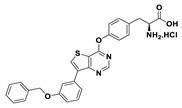	92%
**15a**	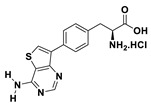	49%
**15b**	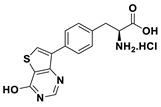	33%
**18a**	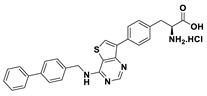	90%

**Table 2:** In vitro test of thienopyrimidine derivatives.

Compound	Structure	TPH1 Inhibition (% Inhibition at 1 µM)	*IC*_50_ (nM)
**pCPA**	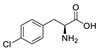	<5% (25% at 100 µM)	
**18a**	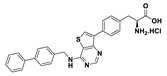	25% (90% at 100 µM)	
**18b**	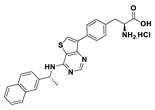	10%	
**18c**	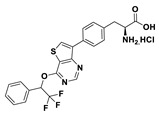	2.5% (10 µM)	
**18d**	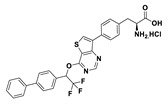	1.2% (10 µM)	
**18e**	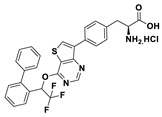	76%	
**18f**	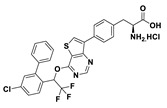	41%	
**18g**	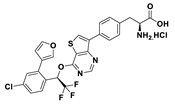	12%	
**18h**	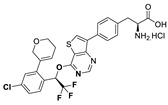	78%	263
**18i**	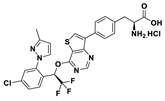	95%	37

**Table 3:** In vitro test of amino thienopyrimidine derivative and prodrugs of compound **18i**.

Compound	Structure	TPH1 Inhibition (% Inhibition at 1 µM)	*IC*_50_ (nM)
**LP533401**		60%	583
**18i**	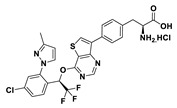	95%	37
**30**	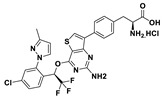	91%	208
**19**	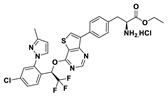	60%	994
**20**	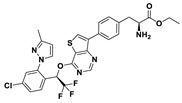	79%	
**21**	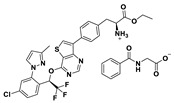	92%	

**Table 4:** PAMPA results.

Compound	Structure	BBB Permeability	
		Pe(10^−6^ cm/s)	logPe
**pCPA**	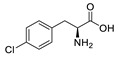	1.85 ^a^	−5.73
**18i**	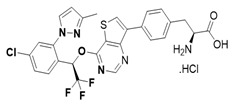	0.00	−10.00

^a^ High permeation predicted Pe (10^−6^ cm/s) > 4.0; low permeation predicted Pe (10^−6^ cm/s) < 2.0.

**Table 5:** Liver microsomal stability, CYP, and Herg inhibition of compound **18i**.

Assay	Result
Liver microsomal stability (rat) ^a^	99% parent remained after 30 min incubation
Liver microsomal stability (human)	99% parent remained after 30 min incubation
CYP inhibition	1A2: 0.1% inhibition at 10 uM
	2C19: 46.5% inhibition at 10 uM
	2D6: 24.6% inhibition at 10 uM
	3A4: 45.3% inhibition at 10 uM
Herg inhibition	<1% inhibition at 10 uM

^a^ Buspirone was used as a positive control (0.1% (rat) 4% (human) remained after 30 min incubation.

**Table 6:** BBB ratio and pharmacokinetic parameters of **18i** in male rats.

Assay	Results
BBB penetration	Plasma: 0.5 h: 2266 ng/mL
	3 h: 1439 ng/mL
	Brain: 0.5 h: BQL ^a^
	3 h: BQL
In vivo PK (iv, 5 mpk)	T_1/2_ (h): 1.85 ± 0.325
	AUCt (μg·h/mL): 1.21 ± 0.058
	AUC∞ (μg·h/mL): 1.22 ± 0.057
	CL (L/h/kg): 4.09 ± 0.186
	Vss (L/kg): 2.16 ± 1.62
(po, 5 mpk, prodrug 21)	T_1/2_ (h): 7.95 ± 0.017
	AUCt (μg·h/mL): 0.189 ± 0.009
	AUC∞ (μg·h/mL): 0.216 ± 0.022
	F = 16%

^a^ below the quantification limit.

## Supplementary Materials

The following are available online at https://www.mdpi.com/article/10.3390/molecules27113417/s1, Experimental Section, Detailed General Procedure for the Synthesis of 6, 9a,b, 11a,b,15a,b, 18a–i, 19, 20, 21, ^1^H NMR and ^13^C NMR, HRMS, LC-MS, HPLC purity and melting point of compounds 6, 9a,b, 11a,b,15a,b, 18a–i, 19, 20, 21 and the sequence of primers for RT-qPCR.

## Abbreviations

BBB, Blood Brain Barriers; pCPA, *para*-chlorophenylalanine; TPH, tryptophan hydroxylase; 5-HT, 5-hydroxytryptamine; CCR2, CC chemokine receptor 2; CCL2, CC chemokine ligand 2; CCR5, CC chemokine receptor 5; TLC, thin layer chromatography; NaH, sodium hydride; DMF, Dimethylformamide; NaOH, sodium hydroxide; KOAc, potassium acetate; DMSO, Dimethyl sulfoxide; EtOAc, ethyl acetate; DCM, dichloromethane; THF, tetrahydrofuran; EtOH, ethanol.
