# Supplementary material for: Identification of New Non-BBB Permeable Tryptophan Hydroxylase Inhibitors for Treating Obesity and Fatty Liver Disease
Source: Molecules. 2022 May 25;27(11):3417. doi: 10.3390/molecules27113417 (PMC9182086; doi:10.3390/molecules27113417)
Supplement: Supplementary file 1 [file molecules-27-03417-s001.zip › molecules-1683509-supplementary.pdf]

Supplementary Material

Identification of New Non-BBB permeable Tryptophan Hydroxylase inhibitors for Treating Obesity and Fatty Liver Disease

Suvarna H. Pagire [1,8,†], Haushabhau S. Pagire [1,8,†], Kun-Young Park [2,3,†], Eun Jung Bae [1], Kwang-eun Kim [2,3], Minhee Kim [1], Jihyeon Yoon [1], Saravanan Parameswaran [1,9], Jun-Ho Choi [1], Sungmi Park [4], Jae-Han Jeon [4,5], Jin Sook Song [7], Myung Ae Bae [7], In-Kyu Lee [4,6], Hail Kim [2,3], Jae Myoung Suh [2,3,*] and Jin Hee Ahn [1,8,*]

[1] Department of Chemistry, Gwangju Institute of Science and Technology, Gwangju 61005, Republic of Korea; shpagire@gist.ac.kr (S.H.P.); hspagire@gist.ac.kr (H.S.P.); helloimej@gist.ac.kr (E.J.B.); kmh0724@gist.ac.kr (M.K.); yjh1@jdbiosci.com (J.Y.); dr.p.saravanan.bi@gmail.com (S.P.); junhochoi@gist.ac.kr (J.-H.C.)
[2] Graduate School of Medical Science and Engineering, Korea Advanced Institute of Science and Technology, Daejeon 34141, Korea; pky1171@kaist.ac.kr (K.-Y.P.); werd2000@snu.ac.kr (K.-e.K.); hailkim@kaist.edu (H.K.)
[3] Biomedical Science and Engineering Interdisciplinary Program, Korea Advanced Institute of Science and Technology, Daejeon 34141, Korea
[4] Leading-edge Research Center for Drug Discovery and Development for Diabetes and Metabolic Disease, Kyungpook National University Hospital, Daegu 41404, Korea; smpark93@gmail.com (S.P.); ggoloo@hanmail.net (J.-H.J.); leei@knu.ac.kr (I.-K.L.)
[5] Department of Internal Medicine, School of Medicine, Kyungpook National University, Kyungpook National University Chilgok Hospital, Daegu 41404, Korea
[6] Department of Internal Medicine, School of Medicine, Kyungpook National University, Kyungpook National University Hospital, Daegu 41944, Korea
[7] Bio and Drug Discovery Division, Korea Research Institute of Chemical Technology, Daejeon 34141, Korea; jssong@krict.re.kr (J.S.S.); mbae@krict.re.kr (M.A.B.)
[8] JD Bioscience, 208 beon-gil, Cheomdangwagi-ro, Buk-gu, Gwangju 61005, Korea
[9] Department of Biotechnology and Bioinformatics, School of Life Sciences, JSS Academy of Higher Education and Research (JSS AHER), Mysuru 570015, India
* Correspondence: jmsuh@kaist.ac.kr (J.M.S.); jhahn@gist.ac.kr (J.H.A.)
† These authors contributed equally to this work.

Experimental Section

1. Chemistry

All reported yields are isolated yields after column chromatography or crystallization. All solvents and chemicals were used as purchased without further purification. ^{1}H NMR spectra and ^{13}C spectra were recorded on a JEOL JNM-ECS400 spectrometer at 400 MHz for ^{1}H NMR and 100 MHz for ^{13}C NMR, respectively. The chemical shift (δ) is expressed in ppm relative to tetramethylsilane (TMS) as an internal standard, and CDCl3, DMSO-d6, and CD3OD were used as solvents. The multiplicity of peaks is expressed as s (singlet), d (doublet), t (triplet), q (quartet), dd (doublet of doublets), td (triplet of doublets), qd (quartet of doublets), dt (doublet of triplets), and m (multiplet). Melting points were determined on a Melting Point M-560, purchased from Buchi. Fast atom bombardment-high-resolution mass spectrometry (FAB-HRMS) data were obtained by a JMS 700 (JEOL, Japan). The purity of all tested compounds was ≥ 95%, as estimated by high performance liquid chromatography (HPLC) analysis. Samples were analyzed on a Waters Agilent HPLC system equipped with a PDA detector and a Waters SB-C18 column (1.8 μm, 2.1 × 50 mm2). The mobile phase was used with buffer A (ultrapure H2O containing 0.1% TFA) and buffer B (chromatographic-grade CH3CN). The flow rate was 0.5 mL/min.

General procedure of thienopyrimidine derivatives 6, 9a, b, 11a, b, and 15a, b

Preparation of ethyl (tert-butoxycarbonyl)-L-tyrosinate (2)

L-tyrosine **1** (15.0 g, 82.79 mmol) was dissolved in 300 mL ethanol and cooled to 0°C. Thionyl chloride (42.04 mL, 0.58 mmol) was added dropwise. The reaction mixture was refluxed under a nitrogen atmosphere for 2 days, and then the solvent was removed under reduced pressure. The reaction mixture was diluted with aqueous $NaHCO_3$, pH 8, and extracted with ethyl acetate. The extracts were washed with brine, dried over anhydrous sodium sulfate, and concentrated in vacuo. After being dried under vacuum, ethyl L-tyrosinate was obtained as a white solid (10.98 g, 63%).

Ethyl L-tyrosinate (10.98 g, 52.473 mmol) was dissolved in methanol (300 mL). The mixture was cooled to 0 °C and trimethylamine (7.314 mL, 52.473 mmol) was added dropwise. A solution of di-tert-butyl dicarbonate (12.597 g, 57.720 mmol) in methanol (50 mL) was added dropwise over 30 min. The mixture was allowed to stand at room temperature for 18 h. The mixture was concentrated, evaporated, diluted with water, and extracted with ethyl acetate. The combined organics were washed with brine and dried over anhydrous sodium sulfate and concentrated in vacuo. The residue was purified by silica gel column chromatography to give ethyl (tert-butoxycarbonyl)-L-tyrosinate **2** as a white solid compound (15.61 g, 96%).

Preparation of ethyl (S)-3-(4-((7-bromothieno[3 ,2-d]pyrimidin-4-yl)oxy)phenyl)-2-((tert-butoxycarbonyl)amino)propanoate (4)

Ethyl (tert-butoxycarbonyl)-L-tyrosinate **2** (247.97 mg, 0.802 mmol) was dissolved in acetonitrile (50 mL). 7-bromo-4-chlorothieno[3,2-d] pyrimidine **3** (200 mg, 0.802 mmol) and cesium carbonate (522.34 mg, 1.603 mmol) were then added. The mixture was heated at 40 °C for 12 h. The reaction mixture was evaporated, diluted with water, extracted with ethyl acetate, and dried with sodium sulfate. The mixture was purified by silica gel column chromatography to give ethyl (S)-3-(4-((7-bromothieno[3,2-d]pyrimidin-4-yl)oxy)phenyl)-2-((tert-butoxycarbonyl)amino) propanoate **4** (365 mg, 87%).

Preparation of (S)-2-amino-3-(4-((7-bromothieno[3,2-d]pyrimidin-4-yl)oxy)phenyl) propanoic acid hydrochloride (6)

NaOH (140 mg, 3.493 mmol) was added to a solution of ethyl (S)-3-(4-((7-bromothieno[3,2-d]pyrimidin-4-yl)oxy)phenyl)-2-((tert-butoxycarbonyl)amino)propanoate **4** (365 mg, 0.699 mmol) in THF/water (80 mL, 3:1). The reaction was stirred at ambient temperature for 12 h. The tetrahydrofuran was removed in vacuo and the resulting solution was

diluted with water and acidified with 35-37% aqueous hydrochloric acid to a pH of 4. The aqueous solution was extracted with ethyl acetate. The combined organic layer was washed with brine, dried over sodium sulfate, and concentrated. (S)-3-(4-((7-bromothieno[3,2-d]pyrimidin-4-yl)oxy)phenyl)-2-((tert-butoxy carbonyl)amino)propanoic acid was obtained (310 mg, 89%).

(S)-3-(4-((7-bromothieno[3,2-d]pyrimidin-4-yl)oxy)phenyl)-2-((tert-butoxycarbonyl)amino) propanoic acid (310 mg, 0.627 mmol) was dissolved in ethyl acetate. To the a mixture, 4M HCl in 1,4-dioxane (5 mL) was added and the mixture was stirred for 12 h. The reaction mixture was concentrated in a vacuum and the residue was collected by filtration to give (S)-2-amino-3-(4-((7-bromothieno[3,2-d]pyrimidin-4-yl)oxy)phenyl)propanoic acid hydrochloride **6** (250 mg, 92%); ^{1}H NMR (400MHz, DMSO-d$_6$) δ 9.40 (s, br, 1H), 8.38 (s, 1H), 8.29 (s, br, 2H), 8.26 (s, 1H), 7.04 (d, *J* = 8.8 Hz, 2H), 6.71 (d, *J* = 8.8 Hz, 2H), 4.01–4.09 (m, 1H), 2.98–3.02 (m, 2H); ^{13}C NMR (100MHz, DMSO-d$_6$): δ 172.27, 163.87,155.68, 155.17,150.43, 135.81,134.11,130.65, 121.84, 116.85, 108.67, 55.50, 35.75; HRMS (FAB) m/z calcd for $C_{15}H_{12}BrN_3O_3S$ [M + H]$^+$ 392.9783, found 393.9858; LC-MS (*m*/*z*): 392.9 [M+H]$^+$; HPLC purity 99.22%; m.p.103–105 °C.

Preparation of ethyl (S)-2-((tert-butoxycarbonyl)amino)-3-(4-((7-(4-hydroxyphenyl) thieno[3,2-d]pyrimidin-4-yl)oxy)phenyl)propanoate (7a)

Ethyl (S)-3-(4-((7-bromothieno[3,2-d]pyrimidin-4-yl)oxy)phenyl)-2-((tert-butoxycarbonyl)amino) propanoate **4** (285 mg, 0.546 mmol) was dissolved in 1,4-dioxane (10 mL). (4-Hydroxyphenyl)boronic acid **5a** (82.77 mg, 0.600 mmol), tetrakis(triphenylphosphine)palladium(0) (31.52 mg, 0.027 mmol), and 2M aqueous potassium carbonate solution (3 mL) were added and the mixture was stirred 2 h at 100 °C. The residue was extracted with water and ethyl acetate and the organic phase was washed with brine. Then, the combined organic fraction was dried over sodium sulfate and filtered. The solvent was removed under reduced pressure to obtain a residue which was purified by silica gel column chromatography (200 mg, 68%).

Ethyl (S)-2-((tert-butoxycarbonyl)amino)-3-(4-((7-(3-hydroxyphenyl) thieno[3,2-d]pyrimidin-4-yl)oxy)phenyl)propanoate (**7b**) was synthesized using a similar method as compound **7a**.

Preparation of (S)-2-amino-3-(4-((7-(4-hydroxyphenyl)thieno[3,2-d]pyrimidin-4-yl)oxy)phenyl)propanoic acid hydrochloride (9a)

This compound was synthesized using a similar method as compound **6**. ^{1}H NMR (400MHz, DMSO-d$_6$) δ 13.82 (s, 1H), 9.66 (s, 1H), 8.83–8.71 (m, 1H), 8.59–8.41 (m, 4H), 7.94 (dd, J= 8.70, 2.14 Hz, 2H), 7.46–7.29 (m, 4H), 6.90 (dd, J= 8.39, 2.14 Hz, 2H), 4.29–4.17 (m, 1H), 3.20 (d, J = 3.66 Hz, 2H); ^{13}C NMR (100MHz, DMSO-d$_6$): δ 170.31, 163.79,160.18, 157.58,154.04, 150.98, 135.68, 132.82,130.95,130.64, 129.27, 124.08, 122.02,118.17, 115.39, 53.14, 35.05; HRMS (FAB) m/z calcd for $C_{21}H_{17}N_3O_4S$ [M + H]$^+$ 407.0940, found 408.1019; LC-MS (*m*/*z*): 408.0 [M+H]$^+$; HPLC purity 98.89%; m.p.228–231 °C.

Preparation of (S)-2-amino-3-(4-((7-(3-hydroxyphenyl)thieno[3,2-d]pyrimidin-4-yl)oxy)phenyl)propanoic acid hydrochloride (9b)

This compound was synthesized using a similar method as compound **6**. (51% yield); ^{1}H NMR (400MHz, DMSO-d$_6$) δ 8.79 (s, 1H), 8.64 (s, 1H), 8.45 (s, br, 2H), 7.55 (s, 1H), 7.46 (d, *J* = 8.4 Hz, 1H), 7.40 (d, *J* = 8.4 Hz, 2H), 7.34 (d, *J* = 8.4 Hz, 2H), 7.29 (t, *J* = 8.0 Hz, 1H), 6.83 (dd, *J* = 8.0, 2.4 Hz, 1H), 4.19–4.27 (m, 1H), 3.16–3.21 (m, 2H); ^{13}C NMR (100MHz, DMSO-d$_6$): δ 170.30, 160.10, 157.49, 154.15, 150.95, 135.67, 134.23, 132.95, 130.93, 130.50, 129.50, 129.32, 122.02, 118.81, 118.26, 115.38, 115.05, 53.12, 35.03; HRMS (FAB) m/z calcd for $C_{21}H_{17}N_3O_4S$ [M + H]$^+$ 407.0940, found 408.1021; LC-MS (*m*/*z*): 408.0 [M+H]$^+$; HPLC purity 97.88%; m.p. 215–216 °C.

Preparation of ethyl (S)-3-(4-((7-(4-(benzyloxy)phenyl)thieno[3,2-d]pyrimidin-4-yl)oxy)phenyl)-2-((tert-butoxycarbonyl)amino)propanoate (10a)

Ethyl (S)-2-((tert-butoxycarbonyl)amino)-3-(4-((7-(4-hydroxyphenyl) thieno[3,2-d]pyrimidin-4-yl)oxy)phenyl)propanoate **7a** (230 mg, 0.254 mmol) was dissolved in anhydrous THF (30 mL). Benzyl alcohol **8** (46.82 mg, 0.254 mmol) and triphenylphosphine (79.99 mg, 0.305 mmol) were added to the reaction mixture. The solution was cooled to 0 °C. Then, diisopropyl azodicarboxylate (0.06 mL, 0.305 mmol) was added dropwise to the reaction mixture. The reaction mixture was stirred under a nitrogen atmosphere for 18 h. The volume was reduced by evaporation and the resulting mixture was added to water followed by extraction with ethyl acetate. The combined organic phase was washed with brine, dried over sodium sulfate, filtered, and the volatiles evaporated in vacuo. Crude product was purified by silica gel column chromatography to give ethyl (S)-3-(4-((7-(4-(benzyloxy)phenyl)thieno[3,2-d]pyrimidin-4-yl)oxy)phenyl)-2-((tert-butoxycarbonyl)amino)propanoate **10a** (140 mg, 98%); ^{1}H NMR (400MHz, $CDCl_3$) δ 8.79 (s, 1H), 8.00 (s, 1H), 7.63–7.65 (m, 1H), 7.31–7.55 (m, 8H), 7.19–7.25 (m, 3H), 7.03 (dd, *J* = 7.6, 2.0 Hz, 1H), 5.15 (s, 2H), 4.55–4.64 (m, 1H), 4.19 (q, *J* = 7.2 Hz, 2H), 3.06–3.21 (m, 2H), 1.44 (s, 9H), 1.26–1.29 (m, 3H).

Preparation of ethyl (S)-3-(4-((7-(3-(benzyloxy)phenyl)thieno[3,2-d]pyrimidin-4-yl)oxy)phenyl)-2-((tert-butoxycarbonyl)amino)propanoate (10b)

This compound was synthesized using a similar method as compound **10a** (128 mg, 53%).

Preparation of (S)-2-amino-3-(4-((7-(4-(benzyloxy)phenyl)thieno[3,2-d]pyrimidin-4-yl)oxy)phenyl)propanoic acid hydrochloride (11a)

This compound was synthesized using a similar method as compound **6**. (23% yield); ^{1}H NMR (400MHz, DMSO-d_6) δ 8.80 (s, 1H), 8.75 (s, 1H), 8.42 (s, br, 1H), 7.84 (s, 1H), 7.70 (d, *J* = 8.4 Hz, 1H), 7.50 (d, *J* = 7.2 Hz, 2H), 7.37–7.44 (m, 5H), 7.31–7.36 (m, 3H), 7.08 (dd, *J* = 8.4, 2.8 Hz, 1H), 5.19 (s, 2H), 4.17–4.22 (m, 1H), 3.15–3.21 (m, 2H); ^{13}C NMR (100MHz, DMSO-d_6): δ 170.40, 160.07, 157.93, 157.48, 156.64, 154.09, 146.45, 136.98, 136.22, 130.93, 130.49, 129.54, 129.24, 128.45, 127.84, 127.69, 126.37, 125.85, 124.67, 124.25, 121.97, 118.17, 115.35, 114.87, 114.71, 69.21, 53.44, 34.88; HRMS (FAB) m/z calcd for $C_{28}H_{23}N_3O_4S$ $[M + H]^+$ 497.1409, found 498.1491; LC-MS (*m*/*z*): 498.1 $[M+H]^+$; HPLC purity 98.79%; m.p. 227–229 °C.

Preparation of (S)-2-amino-3-(4-((7-(4-(benzyloxy)phenyl)thieno[3,2-d]pyrimidin-4-yl)oxy)phenyl)propanoic acid hydrochloride (11b)

This compound was synthesized using a similar method as compound **6**. (66% yield); ^{1}H NMR (400MHz, DMSO-d_6) δ 8.79 (s, 1H), 8.60 (s, 1H), 8.42 (s, br, 2H), 8.06 (d, *J* = 8.8 Hz, 2H), 7.48 (d, *J* = 7.2 Hz, 2H), 7.39–7.43 (m, 4H), 7.32–7.39 (m, 3H), 7.16 (d, *J* = 8.8 Hz, 2H), 5.19 (s, 2H), 4.20–4.27 (m, 1H), 3.16–3.20 (m, 2H); ^{13}C NMR (100MHz, DMSO-d_6): δ 170.34, 163.82, 160.05, 158.46, 154.23, 150.91, 137.03, 135.07, 134.35, 133.035, 132.95, 130.95, 129.66, 128.47, 127.84, 122.01, 120.50, 118.28, 114.77, 69.34, 53.20, 35.09; HRMS (FAB) m/z calcd for $C_{28}H_{23}N_3O_4S$ $[M + H]^+$ 497.1409, found 498.1491; LC-MS (*m*/*z*): 498.1 $[M+H]^+$; HPLC purity 98.37%; m.p. 231–233 °C.

Preparation of ethyl (S)-2-((tert-butoxycarbonyl)amino)-3-(4-(4,4,5,5-tetramethyl-1,3,2-dioxaborolan-2-yl)phenyl)propanoate (12)

To a solution of ethyl (tert-butoxycarbonyl)-L-tyrosinate **2** (44 g, 0.142 mol) and pyridine (44.8 mL, 0.569 mol) in DCM, Triflic anhydride (25.1 mL, 0.149 mol) was added dropwise at 0°C. The reaction mixture was allowed to warm to ambient temperature and the mixture was stirred for an additional 1 h. The reaction mixture was poured into a saturated bicarbonate solution and was extracted with DCM. The organic phase was washed with brine and dried over anhydrous Na_2SO_4. The solvent was removed under reduced pressure and the residue was purified on silica gel to give ethyl (S)-2-((tert-butoxycarbonyl)amino)-3-(4-(((trifluoromethyl)sulfonyl)oxy)phenyl) propanoate (60 g, 95%) as a white solid.

A stirred mixture of ethyl (S)-2-((tert-butoxycarbonyl)amino)-3-(4-(((trifluoromethyl)sulfonyl)oxy)phenyl)propanoate (10 g, 22.67 mmol) in DMSO (40 mL) was added to Bis(pinacolato)diboron (5.75 g, 22.67 mmol) and potassium acetate (6.675 g, 68.01 mmol). The resulting reaction mixture was warmed to 40°C and de-gassed. $PdCl_2dppf.CH_2Cl_2$ (331.75 mg, 0.453 mmol) was charged and the mixture was further de-gassed. The reaction mixture was heated to 100°C for 12 h and then left at room temperature overnight. The mixture was then extracted with diethyl ether (4 X 200 mL). The combined organics were washed with water and brine, dried over anhydrous Na_2SO_4, filtered, and concentrated. The residue was purified by silica gel column chromatography to give ethyl (S)-2-((tert-butoxycarbonyl)amino)-3-(4-(4,4,5,5-tetramethyl-1,3,2-dioxaborolan-2-yl)phenyl)propanoate 12 (8 g, 84%) as a white solid. ^{1}H NMR (400 MHz, DMSO-d_6) : δ 7.58 (d, J = 7.63 Hz, 2H), 7.27 (d, J = 8.70 Hz, 1H), 7.24 (d, J = 7.63 Hz, 2H), 4.16–3.98 (m, 3H), 3.03–2.80 (m, 3H), 1.32 (s, 9H), 1.27 (s, 12H), 1.12 (t, J = 7.02 Hz, 3H).

Preparation of (S)-2-amino-3-(4-(4-aminothieno[3,2-d]pyrimidin-7-yl)phenyl)propanoic acid hydrochloride (15a)Preparation of 7-bromothieno[3,2-d]pyrimidin-4-amine (13a)

7-bromo-4-chlorothieno[3,2-d]pyrimidine **3** (200 mg, 0.802 mmol) was dissolved in isopropyl alcohol (50 mL). A 2M ammonia solution in isopropanol (3 mL) was added to the mixture and stirred overnight at room temperature. After completion of the reaction using brine, the reaction mixture was extracted twice with ethyl acetate. The collected organic layer was dried over anhydrous sodium sulfate and concentrated under reduced pressure to obtain a residue which was purified by column chromatography to obtain the 7-bromothieno[3,2-d]pyrimidin-4-amine **13a** (105 mg, 56%).

Preparation of ethyl (S)-3-(4-(4-aminothieno[3,2-d]pyrimidin-7-yl)phenyl)-2-((tert-butoxy carbonyl)amino) propanoate (14a)

To a mixture of 7-bromothieno[3,2-d]pyrimidin-4-amine (90 mg, 0.391 mmol) in 1,4-dioxane was added ethyl (S)-2-((tert-butoxycarbonyl)amino)-3-(4-(4,4,5,5-tetramethyl-1,3,2-dioxaborolan-2-yl)phenyl)propanoate **12** (180.43 mg, 0.430 mmol), tetrakis(triphenylphosphine)palladium(0) (22.60 mg, 0.020 mmol) and 2M aqueous potassium carbonate solution (2 mL); the mixture was then stirred at 90 °C for 2h.The residue was extracted with water, ethyl acetate, and organic phase were washed with brine. Then, the combined organic fraction was dried over sodium sulfate and filtered. The solvent was removed under reduced pressure to afford a residue which was purified by silica gel column chromatography to give ethyl (S)-3-(4-(4-aminothieno[3,2-d]pyrimidin-7-yl)phenyl)-2-((tert-butoxy carbonyl)amino) propanoate **14a** (120 mg, 69%).

Preparation of (S)-2-amino-3-(4-(4-aminothieno[3,2-d]pyrimidin-7-yl)phenyl)propanoic acid hydrochloride (15a)

This compound was synthesized using a similar method as compound **6**. (70 mg, 74% yield); ^{1}H NMR (400 MHz, DMSO-d_6) : δ 13.86 (s, 1H), 8.87 (s, 2H), 8.64–8.40 (m, 5H), 7.83 (s, 2H), 7.42 (s, 2H), 4.27–4.13 (m, 1H), 3.26–3.13 (m, 2H); ^{13}C NMR (100MHz, DMSO-d_6): δ 170.16, 148.43, 135.33, 134.82, 134.22, 131.13, 129.94, 128.47, 115.71, 53.13, 35.34; HRMS (FAB) m/z calcd for $C_{15}H_{14}N_4O_2S$ $[M + H]^+$ 314.08, found 315.0918; LC-MS (*m*/*z*): 315.0 $[M+H]^+$; HPLC purity 99.27%; m.p. 175–177 °C.

Preparation of (S)-2-amino-3-(4-(4-hydroxythieno[3,2-d]pyrimidin-7-yl)phenyl) propanoic acid hydrochloride (15b)

Preparation of 7-bromothieno[3,2-d]pyrimidin-4-ol (13b)

7-bromo-4-chlorothieno[3,2-d]pyrimidine **3** (200 mg, 0.802 mmol) dissolved in 1,4-dioxane (50 mL). NaOH (64.13 mg, 1.603 mmol) in H_2O was added to the mixture and stirred overnight at room temperature. After completion of the reaction using brine, the reaction mixture was extracted twice with ethyl acetate. The collected organic layer was dried over anhydrous sodium sulfate and concentrated under reduced pressure to obtain

a residue which was purified by column chromatography to obtain the 7-bromothieno[3,2-d]pyrimidin-4-ol (150 mg, 81%).

Preparation of (S)-2-amino-3-(4-(4-hydroxythieno[3,2-d]pyrimidin-7-yl)phenyl)propanoic acid hydrochloride (15b)

This compound was synthesized using a similar method as compound **15a**. (80 mg, 67% yield); ^{1}H NMR (400 MHz, DMSO-d_6) : δ 12.73 (s, 1H), 8.49 (bs, 3H), 8.41 (s, 1H), 8.25 (s, 1H), 7.93 (d, J= 8.24 Hz, 2H), 7.38 (d, J= 8.24 Hz, 2H), 4.25–4.15 (m, 1H), 3.19 (d, J= 6.10 Hz, 2H); ^{13}C NMR (100MHz, DMSO-d_6): δ 170.27, 157.50, 154.73, 146.67, 136.30, 134.60, 132.57, 131.00, 129.71, 128.18, 124.44, 53.17, 35.42; HRMS (FAB) m/z calcd for $C_{15}H_{13}N_3O_3S$ $[M + H]^+$ 315.07, found 316.0758; LC-MS (*m*/*z*): 316.0 $[M+H]^+$; HPLC purity 99.06%; m.p. 294–295 °C.

Preparation of ethyl (S)-3-(4-(4-(([1,1′-biphenyl]-4-ylmethyl)amino)thieno[3,2-d]pyrimidin-7-yl)phenyl)-2-((tert-butoxycarbonyl)amino)propanoate (17a)

7-bromo-4-chlorothieno[3,2-d]pyrimidine **3** (218 mg, 0.873 mmol) and [1,1′-biphenyl]-4-ylmethanamine **16a** (160 mg, 0.873 mmol) were dissolved in 1,4 dioxane. Cesium carbonate (570 mg, 1.746 mmol) was added to the mixture and stirred overnight at room temperature. After completion of the reaction using brine, the reaction mixture was extracted twice with ethyl acetate. The collected organic layer was dried over anhydrous sodium sulfate and concentrated under reduced pressure to obtain a residue which was purified by column chromatography to obtain the title compound N-([1,1′-biphenyl]-4-ylmethyl)-7-bromothieno[3,2-d]pyrimidin-4-amine (300 mg, 87%).

N-([1,1′-biphenyl]-4-ylmethyl)-7-bromothieno[3,2-d]pyrimidin-4-amine (1.5 g, 2.978 mmol) was added to 1,4-dioxane (25 mL), and ethyl (S)-2-((tert-butoxycarbonyl)amino)-3-(4-(4,4,5,5-tetramethyl-1,3,2-dioxaborolan-2-yl)phenyl)propanoate **12** (1.37 g, 3.26 mmol)), tetrakis(triphenylphosphine)palladium(0) (172.06 mg, 0.149 mmol), potassium carbonate (823.11 mg, 5.96 mmol), and water (8 mL) were sequentially added thereto while stirring. The reaction mixture was heated to 90°C, and stirred at 90°C for 3 h. After completion of the reaction using brine, the reaction mixture was extracted twice with ethyl acetate. The collected organic layer was dried over anhydrous sodium sulfate and concentrated under reduced pressure to obtain a foamy residue which was purified by column chromatography to obtain the title compound ethyl (S)-3-(4-(4-(([1,1′-biphenyl]-4-ylmethyl)amino)thieno[3,2-d]pyrimidin-7-yl)phenyl)-2-((tert-butoxycarbonyl)amino)propanoate **17a** (1.5g, 70%).

Preparation of (S)-3-(4-(4-(([1,1′-biphenyl]-4-ylmethyl)amino)thieno[3,2-d]pyrimidin-7-yl)phenyl)-2-aminopropanoic acid hydrochloride (18a)

The title compound was synthesized according to the similar procedure of preparing compound **6.** (200 mg, 74% yield); ^{1}H NMR (400 MHz, DMSO-d_6): δ 8.66 (d, J = 3.21 Hz, 1H), 8.52 (bs, 3H), 8.44 (d, J = 4.12 Hz, 1H), 7.82 (d, J = 7.33 Hz, 2H), 7.68–7.60 (m, 4H), 7.52–7.40 (m, 6H), 7.39–7.32 (m, 1H), 4.88 (s, 2H), 4.27–4.16 (m, 1H), 3.21 (d, J= 3.21 Hz, 2H); ^{13}C NMR (100MHz, DMSO-d_6): δ 170.16, 150.55, 139.81, 139.21, 137.06, 135.60, 133.87, 133.09, 130.73, 130.07, 128.94, 128.64, 128.24, 127.42, 126.77, 126.60, 116.24, 53.15, 43.98, 35.33; HRMS (FAB) m/z calcd for $C_{28}H_{24}N_4O_2S$ $[M + H]^+$ 480.1620, found 481.1695; LC-MS (*m*/*z*): 481.1 $[M+H]^+$; HPLC purity 93.07%; m.p. 251–252 °C.

Preparation of (S)-2-amino-3-(4-(4-(((R)-1-(naphthalen-2-yl)ethyl)amino)thieno[3,2-d]pyrimidin-7-yl)phenyl)propanoic acid hydrochloride (18b)

The title compound was synthesized according to the similar procedure of preparing compound **18a** by using (R)-1-(naphthalen-2-yl)ethan-1-amine **16b.** (130 mg, 77% yield); ^{1}H NMR (400 MHz, DMSO-d_6): δ 8.60 (s, 1H), 8.50 (bs, 3H), 8.44 (s, 1H), 7.96–7.85 (m, 4H), 7.80 (d, J = 7.33 Hz, 2H), 7.65 (d, J = 8.24 Hz, 1H), 7.53–7.44 (m, 3H), 7.41 (d, J = 7.79 Hz, 2H), 5.84–5.74 (m, 1H), 4.25–4.15 (m, 1H), 3.56 (s, 3H), 3.20 (d, J= 6.41 Hz, 2H); ^{13}C NMR (100MHz, DMSO-d_6): δ 170.23, 150.83, 140.83, 135.53, 132.82, 132.25, 130.91, 130.06, 128.64,

128.18, 127.76, 127.53, 126.30, 125.92, 125.03, 124.50, 66.38, 53.16, 35.38, 21.74; HRMS (FAB) m/z calcd for $C_{27}H_{24}N_4O_2S$ $[M + H]^+$ 468.1620, found 469.1698; LC-MS (*m/z*): 469.1 $[M+H]^+$; HPLC purity 93.35%; m.p. 120–122 °C.

General procedure for preparation of 1-([1,1′-biphenyl]-4-yl)-2,2,2-trifluoroethan-1-ol (16d)

Preparation of 1-(4-bromophenyl)-2,2,2-trifluoroethan-1-ol (23d)

4-bromobenzaldehyde **22d** (1 g, 5.405 mmol) and (trifluoromethyl)trimethylsilane (1.921 g, 13.512 mmol) was dissolved in THF. The reaction mixture was cooled to 0 °C in an ice water bath and stirred for ten minutes under a nitrogen atmosphere. Cesium fluoride (2.052 g, 13.512 mmol) was added to the mixture and the solution was stirred for six hours at room temperature. After completion of the reaction using brine, the reaction mixture was extracted twice with ethyl acetate. The collected organic layer was dried over anhydrous sodium sulfate and concentrated under reduced pressure to obtain a residue which was purified by column chromatography to obtain the title compound 1-(4-bromophenyl)-2,2,2-trifluoroethan-1-ol **23d** (1.2 g, 87% yield).

Preparation of 1-([1,1′-biphenyl]-4-yl)-2,2,2-trifluoroethan-1-ol (16d)

1-(4-bromophenyl)-2,2,2-trifluoroethan-1-ol **23d** (1 g, 3.921 mmol), phenyl boronic acid **24a** (525.91 mg, 4.313 mmol), 2M aqueous sodium bicarbonate (658.80 g, 7.842 mmol), and $Pd(PPh_3)_4$ (226.56 mg, 0.196 mmol) were mixed in dioxane (20 mL). The reaction mixture was subsequently irradiated in a single-mode microwave instrument (Biotage Initiator **2.5**) at 90°C for 30 min. The reaction mixture was poured into brine and extracted with ethyl acetate. The combined organic layer was then dried over anhydrous sodium sulfate and concentrated in vacuo. The residue was purified by silica gel column chromatography to give title compound *1-([1,1′-biphenyl]-4-yl)-2,2,2-trifluoroethan-1-ol* **16d** (850 mg, 85%).

The compounds ***23c*** and ***16e-h*** were synthesized according to the similar procedure of preparing compound **16d.**

Preparation of ethyl (2S)-2-((tert-butoxycarbonyl)amino)-3-(4-(4-(2,2,2-trifluoro-1-phenylethoxy)thieno[3,2-d]pyrimidin-7-yl)phenyl)propanoate (17c)

2,2,2-trifluoro-1-phenylethan-1-ol **23c** (300 mg, 1.703 mmol) was dissolved in 4 mL of N,N-dimethylformamide and cooled to 0 °C. Sodium hydride 60% in oil (44.96 mg, 1.874 mmol) was added to the mixture and stirred for 60 min. 7-bromo-4-chloro-thieno[3,2-d]pyrimidine **3** (446.21 mg, 1.788 mmol) was added to the mixture, heated to room temperature, and stirred for 12 hr. The resulting mixture was quenched in aqueous ammonium chloride and extracted twice with ethyl acetate. The organic layer was washed with water and brine, dried over anhydrous sodium sulfate, and concentrated under a reduced pressure to obtain a bubble residue. The resulting residue was purified by column chromatography to obtain the title compound 7-bromo-4-(2,2,2-trifluoro-1-phenylethoxy)thieno[3,2-d]pyrimidine (552 mg, 83%).

7-bromo-4-(2,2,2-trifluoro-1-phenylethoxy)thieno[3,2-d]pyrimidine (550 mg, 1.413 mmol) was added to 1,4-dioxane (25 mL), and ethyl (S)-2-((tert-butoxycarbonyl)amino)-3-(4-(4,4,5,5-tetramethyl-1,3,2-dioxaborolan-2-yl)phenyl)propanoate **12** (651.85 mg, 1.555 mmol)), tetrakis(triphenylphosphine)palladium(0) (81.65 mg, 0.071 mmol), potassium carbonate (390.62 mg, 2.826 mmol), and water (8 mL) were sequentially added thereto while stirring. The reaction mixture was heated to 90°C and stirred at 90°C for 3 h. After completion of the reaction using brine, the reaction mixture was extracted twice with ethyl acetate. The collected organic layer was dried over anhydrous sodium sulfate and concentrated under reduced pressure to obtain a foamy residue which was purified by column chromatography to obtain the title compound ethyl (2S)-2-((tert-butoxycarbonyl)amino)-3-(4-(4-(2,2,2-trifluoro-1-phenylethoxy)thieno[3,2-d]pyrimidin-7-yl)phenyl)propanoate **17c** (572 mg, 79%).

Preparation of (2S)-2-amino-3-(4-(4-(2,2,2-trifluoro-1-phenylethoxy)thieno[3,2-d]pyrimidin-7-yl)phenyl)propanoic acid hydrochloride (18c)

The title compound was synthesized according to the similar procedure of preparing compound **6.** (200 mg, 74% yield); ^{1}H NMR (400 MHz, DMSO-d$_6$) : δ 13.86 (s, 1H), 8.86 (s, 1H), 8.71(s, 1H), 8.42 (bs, 3H), 8.03 (d, J= 7.63 Hz, 2H), 7.75–7.62 (m, 2H), 7.54–7.37 (m, 5H), 7.19 (q, J= 7.02 Hz, 1H), 4.28–4.17 (m, 1H), 3.18 (d, J= 6.10 Hz, 2H); ^{13}C NMR (100MHz, DMSO-d$_6$): δ 170.28, 161.67, 159.97, 153.90, 135.33, 134.99, 133.24, 131.90, 130.98, 130.07, 129.81, 128.92, 128.18, 127.79, 117.97, 53.11, 35.43; HRMS (FAB) m/z calcd for $C_{23}H_{18}F_3N_3O_3S$ [M + H]$^+$ 473.1021, found 474.1098; LC-MS (*m/z*): 474.0 [M+H]; HPLC purity 92.05%; m.p. 242–244 °C.

Preparation of (2S)-3-(4-(4-(1-([1,1′-biphenyl]-4-yl)-2,2,2-trifluoroethoxy)thieno[3,2-d]pyrimidin-7-yl)phenyl)-2-aminopropanoic acid hydrochloride (18d)

The title compound was synthesized according to the similar procedure of preparing compound **18c** by using 1-([1,1′-biphenyl]-4-yl)-2,2,2-trifluoroethan-1-ol **16d.** (135 mg, 82% yield) ^{1}H NMR (400 MHz, DMSO-d$_6$) : δ 8.86 (s, 1H), 8.75–8.69 (m, 1H), 8.05 (t, J= 8.24 Hz, 2H), 7.77 (s, 4H), 7.67 (d, J= 7.93 Hz, 2H), 7.51–7.33 (m, 5H), 7.25 (q, J= 6.71 Hz, 1H), 4.24–4.09 (m, 1H), 3.24–3.09 (m, 2H); ^{13}C NMR (100MHz, DMSO-d$_6$): δ 168.94, 161.65, 159.95, 153.90, 141.82, 139.20, 135.33, 133.18, 131.81, 129.99, 129.77, 128.99, 128.38, 128.13, 127.92, 127.21, 126.83, 117.95, 53.09, 35.52; HRMS (FAB) m/z calcd for $C_{29}H_{22}F_3N_3O_3S$ [M + H]$^+$ 549.1334, found 550.1413; LC-MS (*m/z*): 549.1 [M+H]$^+$; HPLC purity 99.26%; m.p. 133–134 °C.

Preparation of (2S)-3-(4-(4-(1-([1,1′-biphenyl]-2-yl)-2,2,2-trifluoroethoxy)thieno[3,2-d]pyrimidin-7-yl)phenyl)-2-aminopropanoic acid hydrochloride (18e)

The title compound was synthesized according to the similar procedure of preparing compound **18c** by using 1-([1,1′-biphenyl]-2-yl)-2,2,2-trifluoroethan-1-ol **16e.** (183 mg, 72% yield) ^{1}H NMR (400 MHz, DMSO-d$_6$) : δ 8.84 (s, 1H), 8.70 (s, 1H), 8.02 (d, J= 8.24 Hz, 2H), 7.76 (d, J= 7.02 Hz, 1H), 7.61–7.45 (m, 7H), 7.41 (d, J= 7.93 Hz, 2H), 7.34 (d, J= 6.71 Hz, 1H), 7.00 (q, J= 6.71 Hz, 1H), 4.21–4.11 (m, 1H), 3.18 (d, J= 5.19 Hz, 2H); ^{13}C NMR (100MHz, DMSO-d$_6$): δ 170.14, 161.16, 159.78, 153.50, 142.73, 139.13, 135.15, 134.99, 133.01, 131.61, 130.45, 130.01, 129.61, 128.84, 128.35, 128.13, 127.95, 127.84, 127.68, 126.79, 117.64, 53.12, 35.31; HRMS (FAB) m/z calcd for $C_{29}H_{22}F_3N_3O_3S$ [M + H]$^+$ 549.1334, found 550.1411; LC-MS (*m/z*): 549.1 [M+H]$^+$; HPLC purity 95.93%; m.p. 218–220 °C.

Preparation of (2S)-2-amino-3-(4-(4-(1-(5-chloro-[1,1′-biphenyl]-2-yl)-2,2,2-trifluoroethoxy)thieno[3,2-d]pyrimidin-7-yl)phenyl)propanoic acid hydrochloride (18f)

The title compound was synthesized according to the similar procedure of preparing compound **18c** by using 1-(5-chloro-[1,1′-biphenyl]-2-yl)-2,2,2-trifluoroethan-1-ol **16f.** (120 mg, 64% yield) ^{1}H NMR (400 MHz, DMSO-d$_6$) : δ 8.85 (s, 1H), 8.70 (s, 1H), 8.02 (d, J= 7.93 Hz, 2H), 7.76 (d, J= 8.54 Hz, 1H), 7.64–7.47 (m, 6H), 7.45–7.31 (m, 3H), 6.93 (q, J= 6.41 Hz, 1H), 4.25–4.06 (m, 1H), 3.23–3.07 (m, 2H); ^{13}C NMR (100MHz, DMSO-d$_6$): δ 170.32, 161.21, 160.02, 153.67, 144.77, 137.88, 135.32, 135.11, 134.89, 133.30, 131.82, 130.27, 129.80, 128.96, 128.66, 128.46, 128.38, 128.15, 127.21, 117.79, 53.23, 35.49; HRMS (FAB) m/z calcd for $C_{29}H_{21}ClF_3N_3O_3S$ [M + H]$^+$ 583.0944, found 584.1019; LC-MS (*m/z*): 584.0 [M+H]$^+$; HPLC purity 97.25%; m.p. 179–181 °C.

Preparation of (S)-2-amino-3-(4-(4-((R)-1-(4-chloro-2-(furan-3-yl)phenyl)-2,2,2-trifluoroethoxy)thieno[3,2-d]pyrimidin-7-yl)phenyl)propanoic acid hydrochloride (18g)

The title compound was synthesized according to the similar procedure of preparing compound **18c** by using (R)-1-(4-chloro-2-(furan-3-yl)phenyl)-2,2,2-trifluoroethan-1-ol **16g.** (80 mg, 76% yield); ^{1}H NMR (400 MHz, DMSO-d$_6$): δ 8.86 (d, J = 1.53 Hz, 1H), 8.71 (s, 1H), 8.08 (s, 1H), 8.01 (d, J = 7.93 Hz, 2H), 7.93 (d, J = 1.53 Hz, 1H), 7.74 (d, J = 8.24 Hz, 1H), 7.55 (dd, J = 8.54, 2.14 Hz, 1H), 7.51(d, J = 2.14 Hz, 1H), 7.41 (d, J = 7.93 Hz, 2H), 7.16 (q, J = 6.41 Hz, 1H), 6.96 (s, 1H), 4.18–4.09 (m, 1H), 3.24–3.10 (m, 2H); ^{13}C NMR (100MHz, DMSO-

d_6): δ 170.34, 161.32, 160.03, 153.83, 144.37, 141.33, 135.82, 135.37, 134.99, 133.32, 131.76, 130.31, 129.84, 129.22, 128.43, 128.15, 127.85, 122.29, 117.83, 111.43, 53.46, 35.56; HRMS (FAB) m/z calcd for $C_{27}H_{19}ClF_3N_3O_4S$ [M + H]$^+$ 573.0737, found 574.0819; LC-MS (*m*/*z*): 574.0 [M+H]$^+$; HPLC purity 94.93%; m.p. 222–224 °C.

Preparation of (S)-2-amino-3-(4-(4-((R)-1-(4-chloro-2-(5,6-dihydro-2H-pyran-3-yl)phenyl)-2,2,2-trifluoroethoxy)thieno[3,2-d]pyrimidin-7-yl)phenyl)propanoic acid hydrochloride (18h)

The title compound was synthesized according to the similar procedure of preparing compound **18c** by using (R)-1-(4-chloro-2-(5,6-dihydro-2H-pyran-3-yl)phenyl)-2,2,2-trifluoroethan-1-ol **16h.** (90 mg, 69% yield); ^1H NMR (400 MHz, DMSO-d_6): δ 8.87–8.82 (m, 1H), 8.73–8.67 (m, 1H), 8.00 (d, J = 8.24 Hz, 2H), 7.69 (d, J = 8.24 Hz, 1H), 7.53–7.35 (m, 4H), 7.24 (q, J = 6.10 Hz, 1H), 6.03 (s, 1H), 4.49 (d, J = 15.87 Hz, 1H), 4.19 (d, J = 16.48 Hz, 1H), 4.10–4.02 (m, 1H), 3.92–3.79 (m, 2H), 3.25–3.08 (m, 2H), 2.37- 2.27 (m, 2H); ^{13}C NMR (100MHz, DMSO-d_6): δ 170.41, 161.33, 160.02, 153.67, 142.61, 135.55, 135.40, 134.88, 134.78, 133.30, 131.71, 129.86, 129.21, 128.41, 128.14, 127.57, 126.22, 117.88, 67.39, 63.10, 53.65, 35.63, 24.70; HRMS (FAB) m/z calcd for $C_{28}H_{23}ClF_3N_3O_4S$ [M + H]$^+$ 589.1050, found 590.1131; LC-MS (*m*/*z*): 590.1 [M+H]$^+$; HPLC purity 96%; m.p. 218–220 °C.

Synthesis of (S)-2-amino-3-(4-(4-((R)-1-(4-chloro-2-(3-methyl-1H-pyrazol-1-yl)phenyl)-2,2,2-trifluoroethoxy)thieno[3,2-d]pyrimidin-7-yl)phenyl)propanoic acid hydrochloride (18i)

Ethyl (S)-2-((tert-butoxycarbonyl)amino)-3-(4-(4-((R)-1-(4-chloro-2-(3-methyl-1H-pyrazol-1-yl)phenyl)-2,2,2-trifluoroethoxy)thieno[3,2-d]pyrimidin-7-yl)phenyl)propanoate (17i)

(R)-1-(4-chloro-2-(3-methyl-1H-pyrazol-1-yl)phenyl)-2,2,2-trifluoroethan-1-ol **16i** (900 mg, 3.096 mmol) was dissolved in 4 mL of N,N-dimethylformamide and cooled to 0°C. Sodium hydride 60% in oil (185.7 mg, 4.644 mmol) was added to the mixture and stirred for 60 min. 7-bromo-4-chloro-thieno[3,2-d]pyrimidine **3** (811.18 mg, 3.25 mmol) was added to the mixture and stirred at room temperature for 12 hr. The resulting mixture was quenched in aqueous ammonium chloride and extracted twice with ethyl acetate. The organic layer was washed with water and brine, dried over anhydrous sodium sulfate, and concentrated under a reduced pressure to obtain a residue. The resulting residue was purified by column chromatography to obtain of the title compound (R)-7-bromo-4-(1-(4-chloro-2-(3-methyl-1H-pyrazol-1-yl)phenyl)-2,2,2-trifluoroethoxy)thieno[3,2-d]pyrimidine (1.5 g, 96%).

(R)-7-bromo-4-(1-(4-chloro-2-(3-methyl-1H-pyrazol-1-yl)phenyl)-2,2,2-trifluoroethoxy)thieno [3,2-d]pyrimidine (1.5 g, 2.978 mmol) was added to 1,4-dioxane (25 mL), and ethyl (S)-2-((tert-butoxycarbonyl)amino)-3-(4-(4,4,5,5-tetramethyl-1,3,2-dioxaborolan-2-yl)phenyl) propanoate **12** (1.37 g, 3.26 mmol)), tetrakis(triphenylphosphine)palladium(0) (172.06 mg, 0.149 mmol), potassium carbonate (823.11 mg, 5.96 mmol), and water (8 mL) were sequentially added thereto while stirring. The reaction mixture was heated to 90°C, and stirred at 90°C for 3 h. After completion of the reaction using brine, the reaction mixture was extracted twice with ethyl acetate. The collected organic layer was dried over anhydrous sodium sulfate and concentrated under reduced pressure to obtain a foamy residue which was purified by column chromatography to obtain the title compound ethyl (S)-2-((tert-butoxycarbonyl)amino)-3-(4-(4-((R)-1-(4-chloro-2-(3-methyl-1H-pyrazol-1-yl)phenyl)-2,2,2-trifluoroethoxy)thieno[3,2-d]pyrimidin-7-yl)phenyl)propanoate **17i** (1.5g, 70%).^1H NMR (400 MHz, DMSO-d_6) :δ 8.70 (s, 1H), 8.67 (s, 1H), 8.24 (d, J =2.44 Hz, 1H), 7.99 (d, J= 8.24Hz, 2H), 7.88–7.78 (m, 2H), 7.70 (d, J =2.14 Hz, 1H), 7.56 (dd, J= 8.54, 2.14 Hz, 1H), 7.34 (d, J =8.24 Hz, 2H), 6.40 (d, J =2.44 Hz, 1H), 4.32–4.25 (m, 1H), 4.11 (q, J= 5.19 Hz, 2H), 3.22–3.06 (m, 2H), 2.27 (s, 3H), 1.33 (s, 9H), 1.09 (t, J= 7.02 Hz, 3H).

(S)-2-Amino-3-(4-(4-((R)-1-(4-chloro-2-(3-methyl-1H-pyrazol-1-yl)phenyl)-2,2,2-trifluoroethoxy)thieno[3,2-d]pyrimidin-7-yl)phenyl)propanoic acid hydrochloride (18i)

NaOH (50.27 mg, 1.26 mmol) was added to a solution of ethyl (S)-2-((tert-butoxycarbonyl)amino)-3-(4-(4-((R)-1-(4-chloro-2-(3-methyl-1H-pyrazol-1-yl)phenyl)-2,2,2-trifluoroethoxy)thieno[3,2-d]pyrimidin-7-yl)phenyl)propanoate **17i** (180 mg, 0.251 mmol) in THF / water (50 mL, 3:1). The reaction mixture was stirred at ambient temperature for 24 h. The THF was removed in vacuo and the resulting solution was acidified with 1N hydrochloric acid to a pH of 4. More water was added (50 mL) and the aqueous solution was extracted with EtOAC (3 x 50 mL). The combined organic layer was washed with brine, dried over sodium sulfate, and concentrated. A crude product was purified by column chromatography to afford the title compound (S)-2-((tert-butoxycarbonyl)amino)-3-(4-(4-((R)-1-(4-chloro-2-(3-methyl-1H-pyrazol-1-yl)phenyl)-2,2,2-trifluoroethoxy)thieno[3,2-d]pyrimidin-7-yl)phenyl)propanoic acid.

Hydrogen chloride 4.0 M solution in 1,4 dioxane (5 mL) was added to a mixture of (S)-2-((tert-butoxycarbonyl)amino)-3-(4-(4-((R)-1-(4-chloro-2-(3-methyl-1H-pyrazol-1-yl)phenyl)-2,2,2-trifluoroethoxy)thieno[3,2-d]pyrimidin-7-yl)phenyl)propanoic acid in ethyl acetate (10 mL) and the mixture was stirred for 12 h. The mixture was concentrated to a minimum volume and the residue was collected by filtration to give (S)-2-amino-3-(4-(4-((R)-1-(4-chloro-2-(3-methyl-1H-pyrazol-1-yl)phenyl)-2,2,2-trifluoroethoxy)thieno[3,2-d]pyrimidin-7-yl)phenyl)propanoic acid hydrochloride **18i** (121mg, 77%) (Over two steps) as an off white solid. ^{1}H NMR (400 MHz, DMSO-d_6) :δ 13.83 (s,1H), 8.70 (d, J =1.22 Hz, 1H), 8.66 (d, J= 1.53Hz, 1H), 8.37 (bs, 3H), 8.24 (s, 1H), 7.98 (d, J =7.32 Hz, 2H), 7.88–7.77 (m, 1H), 7.72–7.68 (m, 1H), 7.56 (dt, J= 8.54,1.22 Hz, 1H), 7.37 (d, J =7.63 Hz, 2H), 6.43–6.39 (m, 1H), 4.24–4.15 (m, 1H), 3.14 (d, J= 6.10 Hz, 2H), 2.27 (s, 3H); ^{13}C NMR (100MHz, DMSO-d_6): δ 170.34, 157.49, 154.71, 149.94, 146.62, 139.73, 136.26, 134.47, 134.03, 132.74, 132.59, 130.95, 130.74, 129.65, 129.47, 128.17, 127.95, 124.97, 124.40, 107.27, 53.10, 35.46, 13.38; HRMS (FAB) m/z calcd for $C_{27}H_{21}ClF_3N_5O_3S$ [M + H]$^+$ 587.1006, found 588.1089; LC-MS (*m/z*): 588.2 [M+H]; HPLC purity 99.12%; m.p. 215–217 °C.

Synthesis of ethyl (S)-2-amino-3-(4-(4-((R)-1-(4-chloro-2-(3-methyl-1H-pyrazol-1-yl)phenyl)-2,2,2-trifluoroethoxy)thieno[3,2-d]pyrimidin-7-yl)phenyl)propanoate hydrochloride (19)

Hydrogen chloride 4.0 M solution in 1,4 dioxane (5 mL) was added to a mixture of ethyl (S)-2-((tert-butoxycarbonyl)amino)-3-(4-(4-((R)-1-(4-chloro-2-(3-methyl-1H-pyrazol-1-yl)phenyl)-2,2,2-trifluoroethoxy)thieno[3,2-d]pyrimidin-7-yl)phenyl)propanoate (500 mg, 0.698 mmol) in ethyl acetate (25 mL) and the mixture was stirred for 12 h. The mixture was concentrated and filtered to give title compound ethyl (S)-2-amino-3-(4-(4-((R)-1-(4-chloro-2-(3-methyl-1H-pyrazol-1-yl)phenyl)-2,2,2-trifluoroethoxy)thieno[3,2-d]pyrimidin-7-yl)phenyl)propanoate hydrochloride **19** (405 mg, 88%) as an off white solid. ^{1}H NMR (400 MHz, DMSO-d_6) : δ 8.75 (d, J = 3.05 Hz, 1H), 8.62 (d, J = 3.05 Hz, 1H), 8.26 (t, J = 2.44 Hz, 1H), 7.96 (dd, J = 7.93, 2.44 Hz, 2H), 7.89–7.82 (m, 2H), 7.72 (t, J = 2.44 Hz, 1H), 7.59 (dt, J = 8.85, 2.44 Hz, 1H), 7.32 (dd, J = 8.24, 2.14 Hz, 2H), 6.44 (t, J = 2.44 Hz, 1H), 4.02 (q, J = 7.02 Hz, 2H), 3.63–3.58 (m, 1H), 2.95–2.77 (m, 2H), 2.30 (s, 3H), 1.13 (t, J = 7.02 Hz, 3H); ^{13}C NMR (100MHz, DMSO-d_6): δ 168.95, 161.37, 159.95, 135.74, 150.48, 140.66, 135.51, 135.18, 134.65, 133.27, 132.71, 131.92, 130.16, 129.75, 128.13, 128.08, 124.63, 123.83, 117.82, 107.82, 61.71, 53.08, 35.66, 13.83, 13.15; HRMS (FAB) m/z calcd for $C_{29}H_{25}ClF_3N_5O_3S$ [M + H]$^+$ 615.1319, found 616.1399; LC-MS (*m/z*): 616.05 [M+H]; HPLC purity 98.8%; m.p. 102–104 °C.

Synthesis of ethyl (S)-2-amino-3-(4-(4-((R)-1-(4-chloro-2-(3-methyl-1H-pyrazol-1-yl)phenyl)-2,2,2-trifluoroethoxy)thieno[3,2-d]pyrimidin-7-yl)phenyl)propanoate (20)

To a mixture of ethyl (S)-2-((tert-butoxycarbonyl)amino)-3-(4-(4-((R)-1-(4-chloro-2-(3-methyl-1H-pyrazol-1-yl)phenyl)-2,2,2-trifluoroethoxy)thieno[3,2-d]pyrimidin-7-yl)phenyl)propanoate (1.2g, 1.676 mmol) in ethyl acetate (20 mL) was added hydrogen chloride 4.0 M solution in 1,4 dioxane (10 mL) and the mixture was stirred for 12 h. The mixture was concentrated and the residue was dissolved in water. Adjusted pH 8 by aqueous ammonia and extracted with ethyl acetate. The combined organic layer dried over anhydrous sodium sulfate and concentrated in vacuo. The residue was purified by silica gel column

chromatography to give title compound ethyl (S)-2-amino-3-(4-(4-((R)-1-(4-chloro-2-(3-methyl-1H-pyrazol-1-yl)phenyl)-2,2,2-trifluoroethoxy)thieno[3,2-d]pyrimidin-7-yl)phenyl)propanoate **20** (910 mg, 88%) as an off white solid. ^{1}H NMR (400 MHz, DMSO-d$_6$) : δ 8.72 (d, J = 3.05 Hz, 1H), 8.64 (d, J = 3.05 Hz, 1H), 8.26 (t, J = 2.44 Hz, 1H), 7.94 (dd, J = 7.93, 2.44 Hz, 2H), 7.89–7.80 (m, 2H), 7.72 (t, J = 2.44 Hz, 1H), 7.59 (dt, J = 8.85, 2.44 Hz, 1H), 7.30 (dd, J = 8.24, 2.14 Hz, 2H), 6.44 (t, J = 2.44 Hz, 1H), 4.04 (q, J = 7.02 Hz, 2H), 3.62–3.55 (m, 1H), 2.96–2.78 (m, 2H), 2.30 (s, 3H), 1.12 (t, J = 7.02 Hz, 3H); ^{13}C NMR (100MHz, DMSO-d$_6$): δ 173.91, 161.34, 159.98, 153.65, 150.46, 140.67, 135.48, 132.68, 130.13, 129.60, 129.50, 127.95, 127.81, 124.63, 123.85, 107.79, 60.25, 55.23, 36.70, 14.01, 13.48; HRMS (FAB) m/z calcd for $C_{29}H_{25}ClF_3N_5O_3S$ [M + H]$^+$ 615.1319, found 616.14; LC-MS (*m/z*): 616.0 [M+H]; HPLC purity 99.24%; m.p. 180–182 °C.

Synthesis of ethyl (S)-2-amino-3-(4-(4-((R)-1-(4-chloro-2-(3-methyl-1H-pyrazol-1-yl)phenyl)-2,2,2-trifluoroethoxy)thieno[3,2-d]pyrimidin-7-yl)phenyl)propanoate hippurate (21)

Hippuric acid (95.98 mg, 0.536 mmol) was added to a solution of ethyl (S)-2-amino-3-(4-(4-((R)-1-(4-chloro-2-(3-methyl-1H-pyrazol-1-yl)phenyl)-2,2,2-trifluoroethoxy)thieno[3,2-d]pyrimidin-7-yl)phenyl)propanoate (330 mg, 0.536 mmol) in ethanol (50 mL). The reaction mixture was stirred at reflux for 12 h and cooled to room temperature. The reaction mixture was then concentrated under reduced pressure to obtain bubble residue. The resulting residue was dissolved in methyl tert-butyl ether (25 mL) and added n-Hexane to form solid which filtered to give ethyl (S)-2-amino-3-(4-(4-((R)-1-(4-chloro-2-(3-methyl-1H-pyrazol-1-yl)phenyl)-2,2,2-trifluoroethoxy)thieno[3,2-d]pyrimidin-7-yl)phenyl)propanoate hippurate **21** as a white solid. ^{1}H NMR (400 MHz, DMSO-d$_6$): δ 8.77 (t, J = 5.49 Hz, 1H), 8.72 (s, 1H), 8.65 (s, 1H), 8.27 (d, J = 2.44 Hz, 1H), 7.95 (d, J = 7.93 Hz, 2H), 7.89 -7.80 (m, 5H), 7.72 (d, J = 2.14 Hz, 1H), 7.59 (dd, J = 8.54, 2.14 Hz, 1H), 7.57–7.44 (m, 4H), 7.31 (d, J = 8.24 Hz, 2H), 6.44 (d, J = 2.14 Hz, 1H), 4.05 (q, J = 7.02 Hz, 2H), 3.90 (d, J = 5.80 Hz, 2H), 3.66–3.58 (m, 1H), 2.96–2.80 (m, 2H), 2.31 (s, 3H), 1.09 (t, 7.02 Hz, 3H); ^{13}C NMR (100MHz, DMSO-d$_6$): δ 171.44, 166.34, 161.36, 160.00, 153.67, 150.48, 140.69. 135.57, 135.51, 133.97, 132.81, 132.71, 131.34, 131.22, 130.16, 129.64, 129.52, 128.34, 128.00, 127.83, 127.22, 124.66, 123.87, 117.78, 107.82, 60.26, 55.30, 41.50, 36.66, 14.03, 13.50; HRMS (FAB) m/z calcd for $C_{29}H_{25}ClF_3N_5O_3S$ [M + H]$^+$ 615.1319, found 615.7; LC-MS (*m/z*): 616.2 [M+H]$^+$; HPLC purity 98.79%; m.p. 87–89 °C.

Preparation of (S)-2-amino-3-(4-(2-amino-4-((R)-1-(4-chloro-2-(3-methyl-1H-pyrazol-1-yl)phenyl)-2,2,2-trifluoroethoxy)thieno[3,2-d]pyrimidin-7-yl)phenyl)propanoic acid hydrochloride (30)

Preparation of 7-bromo-2,4-dichlorothieno[3,2-d]pyrimidine (27)

A mixture of methyl 3-amino-2-thiophenecarboxylate **25** (2 g, 12.73 mmol) and urea (4.6 g, 76.34 mmol) was heated at 190°C for 4 h in a sealed tube. The hot reaction mixture was poured onto sodium hydroxide solution and any insoluble material was removed by filtration. The mixture was then acidified (HCl, 2N) to yield 1H-thieno[3,2-d]pyrimidine-2,4-dione as an off-white precipitate, which was collected by filtration and air dried (1.1g, 51%). ^{1}H NMR 400 MHz, d$_6$-DMSO) 6.90 (1H, d, J = 5.2Hz), 8.10 (1H, d, J = 5.2Hz), 11.60–11.10 (2H, br s).

To a solution of 1H-thieno[3,2-d]pyrimidine-2,4-dione (1 g, 5.946 mmol) in glacial acetic acid (20 mL), bromine (1.1 mL) was added dropwise. The reaction mixture solution was stirred at 90 °C for 12 h, cooled to room temperature, and then slowly added to ice water (200 mL). The white precipitate was collected and washed several times with water. Air dried the solid 7-bromothieno[3,2-d]pyrimidine-2,4(1H,3H)-dione **26** (1.2 g, 81%), used next step without further purification. ^{1}H NMR (400 MHz, DMSO-d$_6$) δ 11.54 (s, 1H), 11.42 (s, 1H), 8.24 (s, 1H).

Phosphoryl chloride (3.4 mL, 36.43 mmol) was added to 7-bromothieno[3,2-d]pyrimidin-2,4(1H,3H)-dione **26** (900 mg, 3.643 mmol), and then N,N-diethylaniline (1.85 mL, 14.57 mmol) was slowly added. The reaction mixture solution was stirred at 110 °C for 24 h. The mixture solution was cooled to room temperature and then slowly added to ice

water (100 mL). Filtered solid and washed thoroughly with water. Dried solid to obtain title compound 7-bromo-2,4-dichlorothieno[3,2-d]pyrimidine **27** (900 mg, 87%). ^{1}H NMR (400 MHz, DMSO-d_6) δ 8.40 (s, 1H).

Preparation of (R)-7-bromo-4-(1-(4-chloro-2-(3-methyl-1H-pyrazol-1-yl)phenyl)-2,2,2-trifluoro-ethoxy)-N-(4-methoxybenzyl)thieno[3,2-d]pyrimidin-2-amine (28)

(R)-1-(4-chloro-2-(3-methyl-1H-pyrazol-1-yl) phenyl)-2,2,2-trifluoroethan-1-ol **16i** (300 mg, 1.03 mmol) was dissolved in 4 mL of N,N-dimethylformamide and cooled to 0°C. Sodium hydride 60% in oil (41.3 mg, 1.03 mmol) was added to the mixture and stirred for 60 min. 7-bromo-2,4-dichlorothieno[3,2-d]pyrimidine **27** (586.83 mg, 2.06 mmol) was added to the mixture, heated to room temperature, and stirred for 12 hr. The resulting mixture was quenched in aqueous ammonium chloride and extracted twice with ethyl acetate. The organic layer was washed with water and brine, dried over anhydrous sodium sulfate, and concentrated under a reduced pressure to obtain a crude residue. The resulting residue was purified by column chromatography to obtain of the title compound (R)-7-bromo-2-chloro-4-(1-(4-chloro-2-(3-methyl-1H-pyrazol-1-yl)phenyl)-2,2,2-trifluoro-ethoxy)thieno[3,2-d]pyrimidine (257 mg, 46%).

Triethyl amine (94 mg, 0.929 mmol) and (4-methoxyphenyl)methanamine (95.59 mg, 0.697 mmol) were added to a solution of (R)-7-bromo-2-chloro-4-(1-(4-chloro-2-(3-methyl-1H-pyrazol-1-yl)phenyl)-2,2,2-trifluoroethoxy)thieno[3,2-d]pyrimidine (250 mg, 0.465 mmol) in Ethanol (10 mL). The reaction mixture was subsequently irradiated in a single-mode microwave instrument (Biotage Initiator **2.5**) at 150 °C for 1 h. The reaction mixture was evaporated and the crude product was purified by silica gel column chromatography to give (R)-7-bromo-4-(1-(4-chloro-2-(3-methyl-1H-pyrazol-1-yl)phenyl)-2,2,2-trifluoro-ethoxy)-N-(4-methoxybenzyl)thieno[3,2-d]pyrimidin-2-amine **28** (290 mg, 98%).

Preparation of (R)-7-bromo-4-(1-(4-chloro-2-(3-methyl-1H-pyrazol-1-yl)phenyl)-2,2,2-trifluoro-ethoxy)thieno[3,2-d]pyrimidin-2-amine(29)

A mixture of (R)-7-bromo-4-(1-(4-chloro-2-(3-methyl-1H-pyrazol-1-yl)phenyl)-2,2,2-trifluoroethoxy)-N-(4-methoxybenzyl)thieno[3,2-d]pyrimidin-2-amine **28** (290 mg, 0.454 mmol) and trifluoroacetic acid (TFA) (20 mL) in a sealed tube was heated 120°C for 24 h. The reaction mixture was evaporated and the crude product was purified by silica gel column chromatography to give ((R)-7-bromo-4-(1-(4-chloro-2-(3-methyl-1H-pyrazol-1-yl)phenyl)-2,2,2-trifluoroethoxy)thieno[3,2-d]pyrimidin-2-amine **29** (230 mg, 97%).

Preparation of (S)-2-amino-3-(4-(2-amino-4-((R)-1-(4-chloro-2-(3-methyl-1H-pyrazol-1-yl)phe-nyl)-2,2,2-trifluoroethoxy)thieno[3,2-d]pyrimidin-7-yl)phenyl)propanoic acid hydro chloride (30)

(*R*)-7-bromo-4-(1-(4-chloro-2-(3-methyl-1H-pyrazol-1-yl)phenyl)-2,2,2-trifluoroeth-oxy)thieno [3,2-d]pyrimidin-2-amine **29** (230 mg, 0.443 mmol) was added to 1,4-dioxane (25 mL) and ethyl (S)-2-((tert-butoxycarbonyl)amino)-3-(4-(4,4,5,5-tetramethyl-1,3,2-diox-aborolan-2-yl)phenyl) propanoate **12** (223.1 mg, 0.532 mmol)), tetrakis(tri-phenylphosphine)palladium(0) (25.62 mg, 0.022 mmol), potassium carbonate (122.56 mg, 0.887 mmol), and water (2 mL) were sequentially added thereto. The reaction mixture was heated to 90 °C and stirred at 90 °C for 2 h. After completion of the reaction using brine, the reaction mixture was extracted twice with ethyl acetate. The collected organic layer was dried over anhydrous sodium sulfate and concentrated under reduced pressure to obtain a foamy residue which was purified by column chromatography to obtain the title compound ethyl (S)-3-(4-(2-amino-4-((R)-1-(4-chloro-2-(3-methyl-1H-pyrazol-1-yl)phe-nyl)-2,2,2-trifluoroethoxy)thieno [3,2-d]pyrimidin-7-yl)phenyl)-2-((tert-butoxycarbonyl) amino)propanoate (280 mg, 86%).

This compound was synthesized using a similar method as compound **6** (195mg, 80%) (over two steps);

^{1}H NMR (400 MHz, DMSO-d_6): δ 8.50 (bs, 3H), 8.43 (s, 1H), 8.24 (d, J = 2.14 Hz, 1H), 8.00 (d,

J = 7.32 Hz, 2H), 7.80 (d, J=8.24 Hz, 1H), 7.69 (d, J = 2.14 Hz, 1H), 7.63 (d, J = 8.54 Hz, 1H),7.56 (q, J = 6.41 Hz, 1H), 7.36 (d, J = 8.24 Hz, 2H), 6.45 (d, J = 2.14 Hz, 1H), 4.30–4.14 (m, 1H), 3.18 (d, J = 5.80 Hz, 2H), 2.32 (s, 3H); ^{13}C NMR (100MHz, DMSO-d_6): δ 170.33, 161.74, 160.63, 150.58, 140.77, 135.39, 134.45, 133.39, 132.77, 132.36, 130.19, 129.65, 128.23, 127.82, 125.09, 124.29, 107.84, 106.82, 53.09, 35.37, 13.48; HRMS (FAB) m/z calcd for $C_{27}H_{22}ClF_3N_6O_3S$ [M + H]$^+$ 602.1115, found 603.1190; LC-MS (*m/z*): 603.1 [M+H]; HPLC purity 99.50%; m.p. 195–197 °C.

2. Biology:

In vitro efficacy test

3T3-L1 cells (American Type Culture Collection, Manassas, VA) were cultured in DMEM supplemented with 10% FCS and 100 mg ml $^{-1}$ P/S in a humidified chamber under 5% atmospheric CO_2 at 37 °C. Two days after reaching confluence, cells were further cultured in adipocyte differentiation medium (DMEM/10% FBS, P/S, 0.5 mM IBMX, 1 μg ml^{-1} insulin, and 1 μM dexamethasone) (day 0). After 2 days, the medium was changed to adipocyte maintenance medium (DMEM/10% FBS, 1 μg ml^{-1} insulin, P/S) (day 2), and the media was changed every 2 days from day 4 to 8. Cells were treated with compound **16e** at 10 or 50 μM concentrations throughout adipocyte differentiation (day 0 thru day 8). On day 8, differentiated 3T3-L1 preadipocytes were harvested for analysis of gene expression.

In vivo efficacy test

All animal study protocols were approved by the Institutional Animal Care and Use Committee at the Korea Advanced Institute of Science and Technology. Male C57BL/6J mice were purchased from SLC (Shizuoka, Japan) and acclimatized for a week. Mice were housed in a humidity and temperature-controlled environment under a 12 hr light-dark cycle with ad libitum access to water and food. After acclimatization, male mice were fed a high-fat diet (HFD, 60% fat calories, Research diet; New Brunswick, NJ) and received daily treatment with TPH1 inhibitors. Intraperitoneal administration of PBS or 300 mg kg^{-1} pCPA (Sigma, St. Louis, MO) was performed concomitant to the start of HFD feeding. Compound **16e** was dissolved in 5% dimethyl sulfoxide (DMSO) (Sigma, St. Louis, MO) and 25% β-cyclodextrin (Sigma, St. Louis, MO). Vehicle or compound **16e** (100 mg kg^{-1}) was injected intraperitoneally for 10 days to mice after two weeks of HFD feeding. Both compounds **18e** and **20e** were prepared in 10% DMSO and 10% Kolliphor EL (Sigma, St. Louis, MO). Vehicle and 100 mg kg^{-1} of compound **18e** or compound **20e** were administered daily by intraperitoneal injection or oral feeding, respectively, concomitant to the initiation of HFD feeding. Body weight was measured daily and mice were sacrificed for harvesting adipose tissue and liver. Tissue samples were fixed in 10% formalin and embedded in paraffin. 5 μm thick sections were prepared from paraffin-embedded blocks for H&E staining. Average adipocyte sizes were measured using AdipoCount software [23].

Liver Oil-red O staining

Fresh liver tissues were flash frozen in O.C.T. compound (Sakura Finetek, Torrance, CA) and 10 μm cryostat sections were collected on glass slides. Sections were allowed to air-dry for 1 hr, rinsed with PBS for 5 min, and washed with distilled water for 5 min. After washing, sections were placed in 30% isopropanol for 5 min, 60% isopropanol for 5 min twice, and then stained in Oil Red O solution (0.5% Oil red O (Sigma, St. Louis, MO) dissolved in 60% isopropanol) for 15 min. The stained sections were rinsed twice with distilled water, mounted, coverslipped, and documented using bright field microscopy.

RNA isolation and real-time quantitative PCR (RT-qPCR)

Total RNA was extracted from 3T3-L1 cells using Trizol reagent (Invitrogen, Carlsbad, CA) and purified using RNeasy Lipid Tissue Mini kit (Qiagen, Hilden, Germany); an aliquot (2 μg) of total RNA was reversed transcribed using the RevertAidTM First Strand cDNA Synthesis Kit (Thermo Fisher Scientific, Waltham, MA) according to the manufacturer's instructions. Real-time PCR was carried out using SYBR Green (Power SYBR Green

PCR Master Mix, Thermo Fisher Scientific) with a ViiA™ 7 Real-Time PCR system (Applied Biosystems, Waltham, MA). Mouse 36B4 gene expression was used as the internal control to normalize gene expression values.

Supplementary table listing primer sequences for RT-qPCR.

Gene	Forward	Reverse
Srebp1c	GCAGATTTATTCAGCTTTGC	CCCTACCGGTCTTCTATCAA
Fasn	ACCTGGTAGACCACTGCATTGAC	CCTGATGAAACGACACATTCTCA
Cd36	TGAGCCTTCACTGTCTGTTGGA	CTGCTACAGCCAGATTCAGAACTG
Pparg	AAGAGCTGACCCAATGGTTG	ACCCTTGCATCCTTCACAAG
Cidea	TGACCCCCCTCATACATCCA	GGCTACTTCGGTCATGGTTTG
Rplp0	ACAGGGCGACCTGGAAG	CTTCCCTCGGAGCGACAT
